# Proximity-based proteomic profiling uncovers distinct interactome of human RAG1 and RAG2: RAG1&RAG2 interaction networks

**DOI:** 10.3724/abbs.2025246

**Published:** 2026-01-27

**Authors:** Junye Hong, Xueming Zheng, Kunyu Wei, Qingyi Lu, Guangrui Huang, Yuhang Zhang

**Affiliations:** 1 School of Life Sciences and Biotechnology Shanghai Jiao Tong University Shanghai 200240 China; 2 Department of Biochemistry and Molecular Biology School of Medicine Jiangsu University Zhenjiang 212013 China; 3 Beijing University of Chinese Medicine Beijing 102488 China; 4 Sheng Yushou Center of Cell Biology and Immunology Shanghai Jiao Tong University Shanghai 200240 China

**Keywords:** biotinylation, mass spectrometry, nuclear transport, protein interaction map, V(D)J recombinase

## Abstract

The recombination-activating gene (RAG) complex initiates adaptive immunity by catalyzing V(D)J recombination to generate diverse antigen receptors. While the catalytic function of the RAG core is well defined, its regulatory interactions and physiological roles remain poorly understood due to limited knowledge of RAG-associated proteins. The RAG complex forms a heterotetramer of two RAG1 and RAG2 subunits, yet the individual contributions of each subunit remain unclear. Here, we use TurboID-mediated proximity labelling to map the human RAG interactome. By fusing TurboID to RAG1 or RAG2, we identify 88 RAG1- and 146 RAG2-associated proteins, with only 23 shared proteins, indicating distinct sets of proximal proteins. Although RAG1 and RAG2 are thought to exert their physiological functions by forming a complex, they display distinct potential interaction networks, suggesting subunit-specific functions and revealing their spatial proximity to each subunit. These findings uncover distinct RAG1 and RAG2 interaction landscapes and establish a framework for exploring broader RAG functions in immunity.

## Introduction

The recombination-activating gene (RAG) complex is a core component of the adaptive immune system, playing a pivotal role in generating antigen receptor diversity [ 1– 3]. RAG specifically recognizes recombination signal sequences (RSS) that flank variable (V), diversity (D), and joining (J) gene segments in immunoglobulin (Ig) and T-cell receptor (TCR) loci [ 2– 4]. It catalyzes site-specific DNA cleavage at RSS-coding sequence junctions, producing double-strand breaks (DSBs) that initiate V(D)J recombination and enable the assembly of a diverse antigen receptor repertoire [ 3, 5] ( Supplementary Figure S1). Evolutionary and biochemical analyses suggest that RAG originates from a transposon-encoded transposase capable of cut-and-paste transposition of mobile genetic elements [ 6– 12]. In jawed vertebrates, this function became increasingly specialized: while RAG lost its transposase activity, it retained DNA endonuclease activity—a shift likely reflecting co-evolution with the host genome and the emergence of a more tightly-regulated immune repertoire [ 11, 13, 14]. Despite decades of investigation into the enzymatic and structural properties of RAG, its regulation within the complex cellular environment remains poorly understood [ 15 , 16]. This gap limits our understanding of RAG’s broader biological roles, especially in humans, including how its activity is modulated in different contexts and whether it may participate in functions beyond V(D)J recombination.

Beyond its catalytic activity, RAG interacts with various host factors to coordinate its function with subsequent DNA repair. For instance, RAG binds to modified histones, influencing chromatin accessibility during V(D)J recombination [ 17– 20]. The RING domain of RAG1 also functions as an E3 ubiquitin ligase, thereby mediating histone ubiquitination and modulating chromatin dynamics [ 21– 24]. Additionally, RAG1 interacts with Vpr-binding protein (VprBP; also known as DCAF1, DDB1- and CUL4-associated factor 1), which targets RAG1 for ubiquitin-mediated degradation, thereby controlling the levels of V(D)J recombination activity under physiological conditions [ 25, 26]. RAG has also been reported to associate with Ku70, a key factor in the non-homologous end joining (NHEJ) DNA repair pathway, to promote efficient DSB repair during V(D)J recombination [27]. While these studies have identified individual RAG-associated factors, the full extent of the RAG interaction network remains elusive. This is likely due to the transient and dynamic nature of many RAG interactions that are hard to capture with conventional biochemical approaches. As a result, comprehensive mapping of the RAG interactome is still lacking.

Proximity labelling techniques such as BioID (proximity-dependent biotin identification) have emerged as powerful tools for investigating intracellular protein-protein interactions, particularly those that are transient or difficult to capture by conventional methods [ 28, 29]. Ryan *et al*. [30] used a biotin ligase (BirM) fused to the N-terminus of murine RAG1 to map RAG-associated proteins, uncovering a complex and dynamic interaction network. However, such work focused on mouse RAG, and the relatively low catalytic efficiency of BirM constrained its ability to label low-affinity or short-lived interactions, thus limiting the completeness of the resulting interactome. To overcome these limitations, we utilized TurboID (highly active, engineered biotin ligase) that enables rapid and efficient protein labelling in living cells [ 31, 32].

Structural studies have shown that the RAG complex assembles as a heterotetramer, with RAG2 positioned atop the catalytic RAG1 dimer, which forms the central scaffold of the complex [33]. Given this spatial arrangement, fusing a biotin ligase exclusively to RAG1 may fail to label interactors associated with more distal regions—particularly those in proximity to RAG2. In addition, a fraction of RAG2 exists in a free state *in vivo*, and interactors associated with this free pool would also absolutely escape detection by a RAG1-fusion BioID approach [ 34, 35]. To achieve a more comprehensive interactome map, we independently fused TurboID to the N-termini of human RAG1 and RAG2. This strategy allowed efficient biotinylation of proximal proteins and revealed striking differences in the interaction networks of RAG1 versus RAG2.

Previous studies of the RAG interactome have primarily focused on mouse models, and information on human RAG-interacting proteins remains sparse. Here, we employed human RAG proteins and a human cell line to more accurately define the human RAG interactome. This human-specific approach provides valuable insights into RAG interactions in our species and will facilitate comparisons between human and mouse RAG proteins in the future.

Functional enrichment analysis of the identified interactors suggests that RAG1 and RAG2 may play distinct regulatory roles. Together, these findings uncover subunit-specific interaction profiles for human RAG1 and RAG2, highlighting previously underappreciated regulatory dimensions of the RAG complex and providing a conceptual framework for dissecting its multifaceted roles in adaptive immunity.

## Materials and Methods

### Plasmids and constructs

All plasmids used in this study are summarized in Supplementary Table S1. Human RAG1 and RAG2 coding sequences were amplified by PCR from the cDNA pool derived from HEK293T cells. For biotin-based proximity labelling experiments, the *TurboID* gene obtained from Addgene (Watertown, USA) was cloned at the N-terminus of either RAG1 or RAG2. Each TurboID-fused construct was co-expressed with its complementary RAG subunit in the mammalian expression vector pTT5 (a gift from Schatz Lab) with the two genes separated by an internal ribosome entry site (IRES), thereby allowing for coordinated bicistronic expression. To facilitate detection, an HA epitope tag was appended to the C-terminus of the TurboID-RAG1 fusion protein for western blot analysis. For GST or MBP pull-down assays, coding sequences of candidate RAG-interacting proteins—including KPNA3 (Karyopherin Subunit Alpha 3), NUP37 (Nucleoporin 37), and NUP133—were amplified from the HEK293T cDNA pool and cloned and inserted into the pTT5 vector with an N-terminal GST tag. The cDNA encoding human *VprBP* was cloned and inserted into the pEBB vector with an N-terminal HA tag. Full-length RAG1 and RAG2 were also cloned and inserted into pTT5, each fused with an N-terminal maltose-binding protein (MBP) tag to enable affinity purification and subsequent protein-protein interaction assays.

### Western blot analysis

Forty-eight hours after transfection with the expression vectors, HEK293T cells were harvested and lysed on ice in buffer containing 50 mM Tris-HCl (pH 7.5), 150 mM NaCl, 1% NP-40, and a protease inhibitor cocktail. To ensure thorough disruption, the lysates were subjected to brief sonication and then centrifuged at high speed to remove insoluble debris. The resulting supernatants were mixed with SDS loading buffer, boiled, and separated by SDS-PAGE. Proteins were transferred onto PVDF membranes (IPVH00010; Merck Millipore, Darmstadt, Germany) and probed with the indicated primary antibodies, including anti-HA (3724S; Cell Signaling Technology, Danvers, USA), anti-RAG2 (a gift from Schatz Lab), anti-GST (66001-2-Ig; Proteintech, Wuhan, China), anti-MBP (M20051; Abmart, Shanghai, China), and anti-β-actin (P30002; Abmart). For protein level detection, HRP-conjugated secondary antibodies, goat anti-rabbit IgG (21002; Sangon Biotech, Shanghai, China) and goat anti-mouse IgG (M21001; Abmart), were used. Streptavidin-HRP conjugate (B110053; Sangon Biotech) was employed to detect biotinylated proteins. Chemiluminescent signals were visualized using standard ECL reagents (36208ES; Yeasen, Shanghai, China). All antibodies utilized are detailed in Supplementary Table S2.

### *In vivo* GST pull-down assays

For *in vivo* GST pull-down assays, HEK293T cells (a gift from Hu Lab) were co-transfected with expression plasmids encoding MBP-tagged RAG1/2 and GST-tagged target proteins according to the experimental design. After 48 h, the cells were harvested and lysed on ice for 30 min in lysis buffer containing 50 mM Tris-HCl (pH 7.5), 150 mM NaCl, 1% NP-40, and a protease inhibitor cocktail. Lysates were clarified by centrifugation at 16,500 *g* for 20 min at 4°C to remove insoluble debris. The resulting supernatants were incubated with glutathione agarose beads (SA008025; Smart-lifesciences, Changzhou, China) at 4°C for 2 h with gentle rotation to capture GST-tagged proteins and their interacting partners. After incubation, the beads were washed three times with lysis buffer to eliminate non-specific binding. Bound proteins were eluted by boiling the beads in SDS loading buffer for 5 min. Eluted samples were analyzed by SDS-PAGE and western blot analysis to evaluate interactions between RAG proteins and the candidate binding partners.

For MBP pull-down assays, an identical procedure was followed, except that dextrin beads (SA077025; Smart-lifesciences) were used to enrich MBP-tagged proteins and their interacting partners.

### Cell culture and transfection

HEK293T cells were maintained in Dulbecco’s modified Eagle’s medium (DMEM, 10027CV; Corning, New York, USA) supplemented with 15% fetal bovine serum (FSS500; ExCell Bio, Shanghai, China) and 1% penicillin-streptomycin-glutamine (10378016; Thermo Fisher Scientific, Waltham, USA) at 37°C in a humidified atmosphere containing 5% CO _2_. Plasmid transfection was performed using PEI-MAX transfection reagent (24765; Polysciences, Warrington, USA) at a plasmid to polyethylenimine (PEI) ratio of 1:3 (w/w). Six hours after transfection, the culture medium was replaced by fresh medium. Expi293F cells (A14527; Thermo Fisher Scientific) were cultured in serum-free medium (P82019; OPM Biosciences, Pleasanton, USA) at 37°C in a humidified incubator with 8% CO _2_ and constantly rotated at 120 rpm. For transfection, PEI-MAX was used at a plasmid-to-PEI ratio of 1:3, with 1–2 μg of plasmid DNA added per milliliter of culture volume.

### *In vivo* recombination assay

RAG1 and RAG2 co-expression plasmid (2 μg each) or MBP-control plasmid (2 μg) were co-transfected with 2 μg of the p290GFP (a gift from Schatz Lab) reporter plasmid into HEK293T cells using PEI-MAX. Six hours after transfection, the culture medium was replaced by fresh medium. Cells were collected 48 h post-transfection, washed twice with PBS containing 1% FBS, and stained with 4′,6-diamidino-2-phenylindole (DAPI) at room temperature for 20 min. The percentage of GFP-positive cells was measured on a CytoFLEX S flow cytometer (Beckman Coulter, Brea, USA) using the FITC channel, and data were analyzed with FlowJo v10.8.1 ( Supplementary Figure S2).

### Biotin ligation assay

For biotin-based proximity labelling, HEK293T cells were transfected with one of the following plasmids: pTT5-TurboID-hRAG1-HA-IRES-hRAG2, pTT5-hRAG1-HA-IRES-TurboID-hRAG2, or pTT5-TurboID-HA. Thirty-six hours post-transfection, the culture medium was replaced by fresh medium containing 50 μM D-biotin (A100340; Sangon Biotech), and the cells were incubated at 37°C for 15 min to allow biotin labelling. The labelling reaction was terminated by placing the cells on ice, followed by two washes with pre-chilled PBS.

Cells were lysed in 4.5 mL of cold lysis buffer (50 mM Tris-HCl, pH 7.4, 150 mM NaCl, 8 M urea, 1% Triton X-100, and protease inhibitor cocktail) on ice for 20 min. Lysates were further disrupted by three cycles of sonication (5 s per cycle) and then centrifuged at 16,500 *g* for 15 min at 4°C. The supernatant was collected, and the residual lysate was incubated with 200 μL of gelatin beads (SA054005; Smart-lifesciences) at 4°C for 2 h to remove potential fibronectin interference. After centrifugation, the supernatant was collected for downstream enrichment.

Cleared lysates were incubated with streptavidin magarose beads (SM007005; Smart-lifesciences) at 4°C for 2 h with gentle rotation to capture biotin-labelled proteins. The beads were washed three times at room temperature with lysis buffer (50 mM Tris-HCl, pH 7.4, 150 mM NaCl, and 8 M urea) under gentle shaking to remove non-specifically bound proteins. On-bead trypsin (20233; Thermo Fisher Scientific) digestion was performed to release bound proteins. The resulting peptides were desalted and purified using Pierce C18 Spin Tips (84850; Thermo Fisher Scientific) and then reconstituted in 0.1% formic acid prior to analysis. Western blot analysis was used to validate the biotinylation efficiency.

### Mass spectrometry and protein identification

Peptides from the on-beads trypsin digestion were analyzed using an Easy-nLC 1200 system coupled to a Q Exactive Plus Orbitrap mass spectrometer (Thermo Fisher Scientific). Prior to analysis, peptides were separated on a C18 reversed-phase analytical column (75 μm × 20 cm, 3 μm particle size) using a linear gradient at a flow rate of 300 nL/min. Mobile phase A consisted of 99.9% H _2_O with 0.1% formic acid, and mobile phase B consisted of 80% acetonitrile with 0.1% formic acid. The elution gradient was performed as follows: 0–3 min, 2%–6% B; 3–92 min, 6%–20% B; 92%–107 min, 20%–32% B; 107–108 min, 32%–100% B; and 108–120 min, 100% B. The mass spectrometer was operated in positive electrospray ionization (ESI+) mode using a data-dependent acquisition (DDA) strategy. Full MS scans were acquired in the Orbitrap with a resolution of 70,000 over an m/z range of 350–1800 (AGC target: 3e6). The top 20 most intense precursor ions (charge state ≥ +1) were selected for higher-energy collisional dissociation (HCD) fragmentation with a normalized collision energy (NCE) of 28.0. MS/MS scans were acquired in the Orbitrap at a resolution of 17,500 (AGC target: 1e5). The maximum injection times were set to 50 ms for MS1 scans and 45 ms for MS2 scans. Dynamic exclusion was enabled with a duration of 30 s. The capillary temperature was 275°C, and the spray voltage was set to 1800 V.

Raw data were processed using Proteome Discoverer software (version 3.0; Thermo Fisher Scientific). Spectra were searched against the human UniProt database using the following parameters: precursor mass tolerance of 10 ppm, fragment mass tolerance of 0.020 Da, and trypsin as the digestion enzyme with up to two missed cleavages allowed. Carbamidomethylation of cysteine was set as a fixed modification, while variable modifications included methionine oxidation, asparagine/glutamine deamidation, N-terminal acetylation (+42.011 Da), methionine loss (–131.040 Da), and methionine loss with N-terminal acetylation (–89.030 Da). Both peptide- and protein-level false discovery rates (FDRs) were controlled at ≤ 1%. For label-free quantification, the Top3 approach was used based on the intensity of the top three unique peptides for each protein. Protein abundances were normalized using a protein abundance-based normalization method. Statistical analysis of quantitative differences was performed using ANOVA at the individual protein level.

### Heat-map generation

To visualize the expression patterns of RAG-interacting genes across various human and murine immune cell types, we performed the following analyses using R (version 4.4) with Bioconductor packages celldex, SummarizedExperiment, and ComplexHeatmap. Two publicly available reference transcriptome datasets were retrieved from the celldex package: HumanPrimaryCellAtlasData (HPCA, representing 37 human primary cell types, including immune and non-immune cells) and ImmGenData (representing murine immune cell populations from the Immunological Genome Project).

The log-normalized expression matrices (logcounts) were extracted from both datasets, with genes as rows and samples as columns. Cell type annotations were accessed via the “label.fine” metadata field.

For the HPCA dataset, we selected the following immune cell subtypes: progenitor B cells, immature B cells, naive B cells, plasma B cells, natural killer cells, dendritic cells and monocytes. For the ImmGen dataset, we selected T-cell subsets spanning early thymic development and peripheral differentiation, including double-negative T cells (T.DN1–T.DN4), double-positive T cells (T.DP), regulatory T cells (T.Tregs), effector T cells (T.EFF), and memory T cells (T.MEM).

For each selected subtype, the average gene expression value was calculated across all samples within that subtype using the rowMeans() function, resulting in a summarized expression profile per subtype. To normalize gene expression values across different cell types, Z score transformation was applied on a per-row basis. Z-score-normalized matrices were visualized using the ComplexHeatmap package. A diverging color gradient (blue-white-red) was used to represent low-to-high relative expression levels of each gene.

### Data analysis

Proteins identified in the TurboID proximity labelling experiments were quantified, and fold change (FC) values were calculated relative to the corresponding control groups. For downstream enrichment analyses, proteins with a fold change greater than 4 (FC > 4) and *P* value < 0.05 were considered significantly enriched. In addition, proteins detected exclusively in the experimental groups—defined as being present in at least two out of three biological replicates but absent in controls—were included as specifically enriched interactors.

The total set of RAG1- and RAG2-enriched proteins (211) was compared with the transcription profiles of bone marrow-derived human pro-B cells (GSE115655) to confirm that the interactome proteins are present under the physiological conditions in which RAG1 and RAG2 are expressed. Additionally, to assess expression in T cells, we utilized single-cell RNA-seq data from human thymic T-cell development obtained from ArrayExpress (accession number: E-MTAB-8581), specifically examining expression at the double-positive (DP) thymocyte stage.

STRING (Search Tool for the Retrieval of Interacting Genes/Proteins) analysis and UniProt keyword enrichment analysis were conducted using the built-in enrichment analysis tool available on STRING (v12.0) at https://string-db.org, with the STRING database used as the reference background.

Gene Ontology (GO) enrichment analyses for RAG1- and RAG2-associated proteins were independently performed using the clusterProfiler R package (v4.12) [ 36, 37]. In the enrichment analysis, false discovery rate (FDR) values were calculated using the Benjamini-Hochberg correction method to adjust for multiple hypothesis testing. GO terms with a *P* value < 0.05 and an FDR < 0.2 were considered statistically significant in this study. Visualization of the enrichment results was carried out using the ggplot2 R package [38] in R 4.4.

## Results

### Structural analysis of the RAG complex and the biotin ligase fusion design

**Figure 1:**
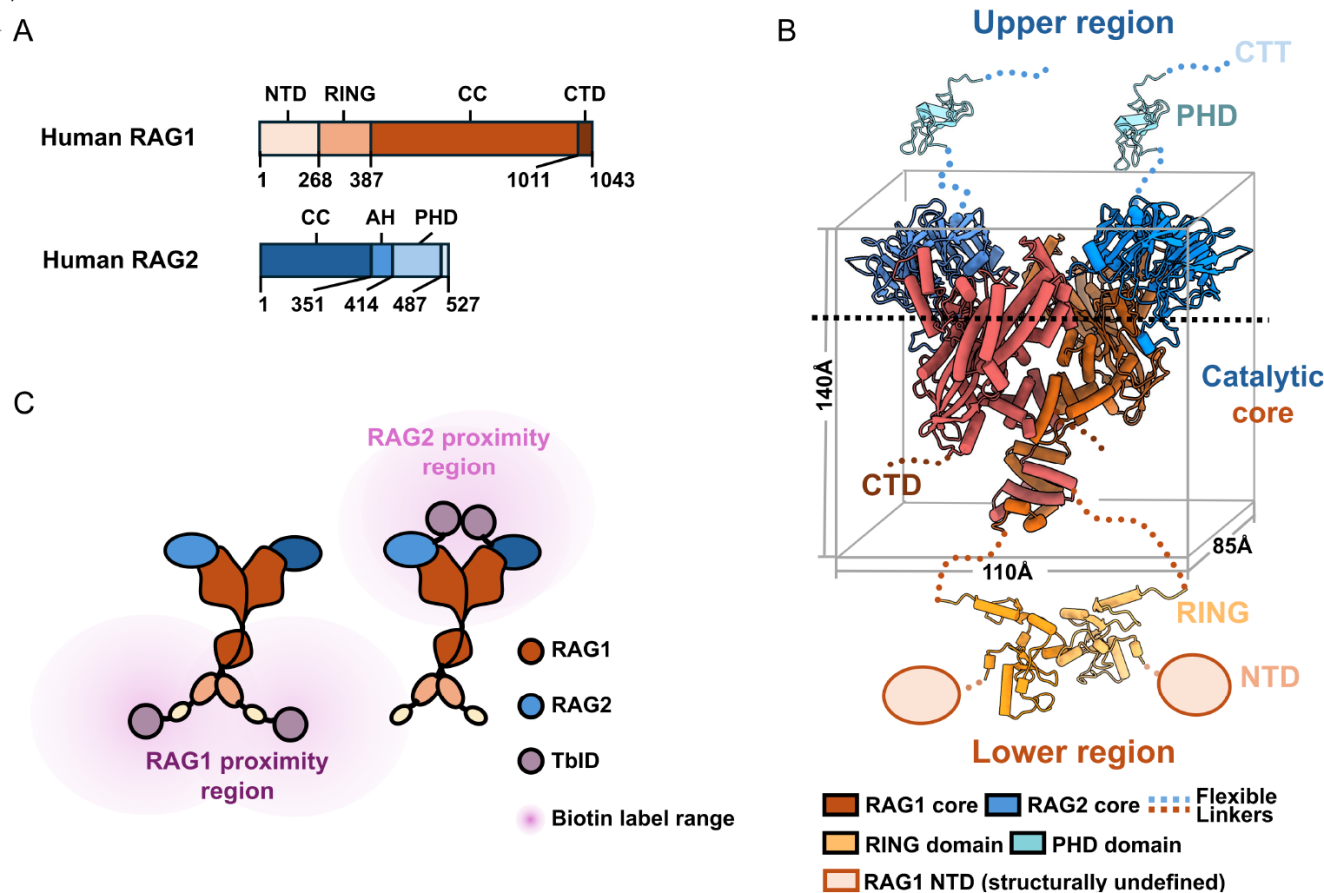
Figure 1 Domain architecture and structure of RAG1/2 (A) Schematic of human RAG1 and RAG2 domain organization. Key domains are labelled, including the catalytic core (CC), N-terminal domain (NTD), really interesting new gene (RING), C-terminal domain (CTD), acidic hinge (AH), and plant homeodomain (PHD) domains. (B) Structure model of the mouse RAG1/2 complex. In the model, solved regions are shown in cartoon mode, whereas unsolved regions are represented by dashed lines and circles. The colors of the corresponding domains are assigned according to the schematic of the corresponding human RAG domains in panel (A). CC, PDB: 4XWW; RING, PDB: 1RMD; PHD, PDB: 2V83. The grey line box demonstrates the three-dimensional space occupied by RAG CC. The black dashed line demarcates the upper and lower regions of the RAG complex. (C) Schematic representation of the biotinylation range following N-terminal TurboID fusion to RAG1 or RAG2. TurboID-RAG1 primarily labels the lower region of the RAG complex, whereas TurboID-RAG2 shows labelling coverage predominantly in the upper region.

The RAG complex forms an ~357 kDa heterotetramer composed of two RAG1 and two RAG2 subunits, each containing multiple functional domains connected by flexible peptide linkers ( Figure 1A). RAG1 residues 384–1008 and RAG2 residues 1–352 constitute the tightly associated catalytic core, which adopts an approximately 110 × 85 × 140 Å structure as resolved by previous studies ( Figure 1B). In this architecture, the RAG1 subunits form a central homodimer that serves as the scaffold, while the two RAG2 subunits occupy the “upper surface” of the complex. The N-termini of RAG1 face downwards, and substantial portions of both proteins—outside the catalytic core—occupy the “lower surface” of the complex [ 3, 33 , 39, 40] ( Figure 1B).

Given BioID’s effective labelling radius of ~10–20 nm [ 41, 42], fusion of biotin ligase only on the RAG1 or RAG2 N-termini likely restricted labelling to a subset of nearby proteins and underrepresented interactors associated with distal RAG2 or distal regions of RAG1 ( Figure 1C). Compounding this spatial limitation, BirM’s relatively low enzymatic activity required extended labelling time (~24 h), reducing its capacity to tag weak or transient interactors [43]. These structural and enzymatic constraints together highlight the need for a more effective proximity labelling strategy to achieve comprehensive mapping of RAG-associated proteins, covering both the “upper region” and “lower region”.

### Subunit-resolved proximity labelling reveals differential biotinylation patterns

**Figure 2:**
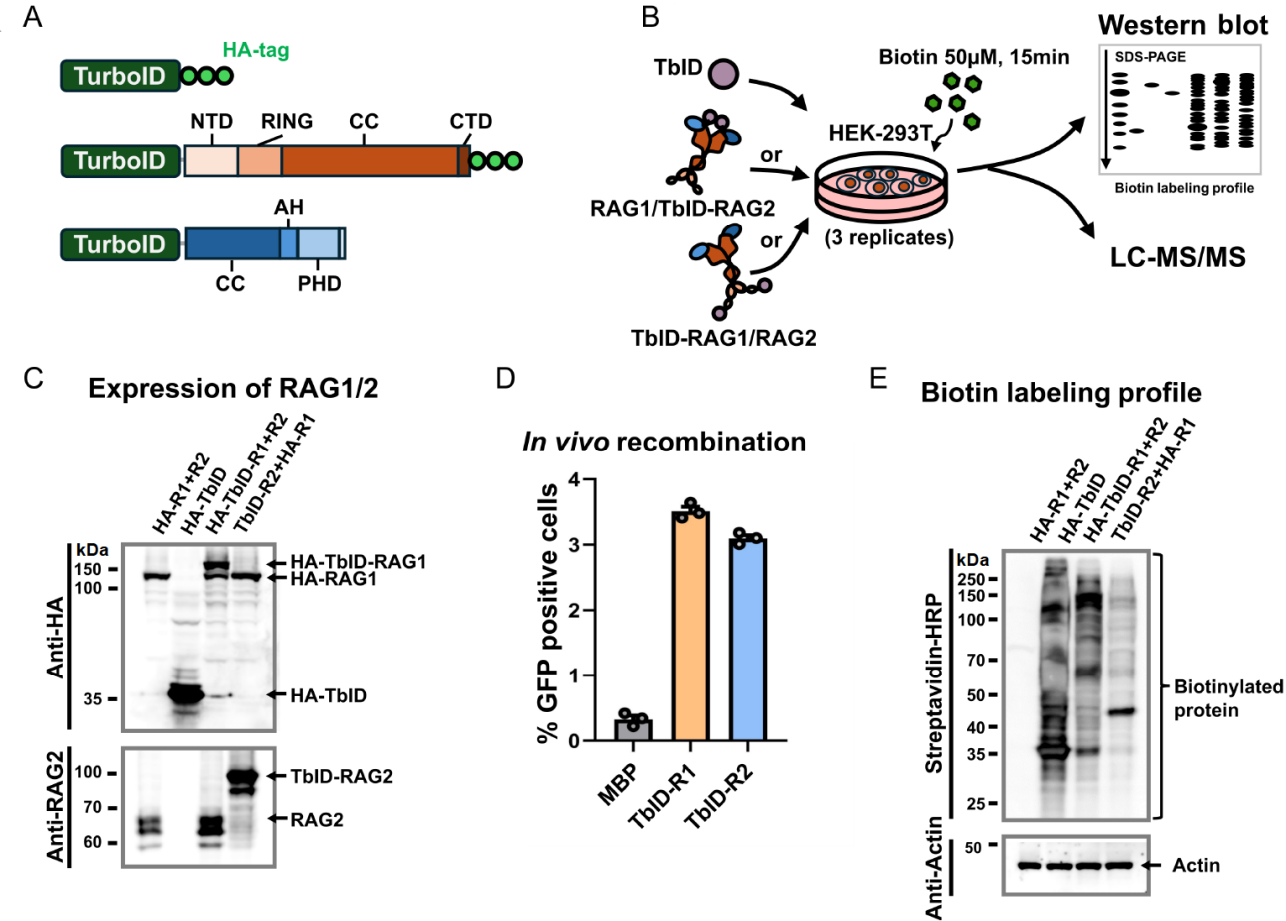
Figure 2 Differential proximity labelling mediated by RAG1 and RAG2 (A) Schematic of TurboID control and TurboID-tagged RAG1 and RAG2 constructs. The diagram illustrates TurboID (green box) expressed alone or fused to the N-terminus of RAG1 or RAG2. Green dots represent HA tags. (B) Schematic of the experiments for biotin proximity labelling mediated by RAG1 and RAG2. (C) Western blot analysis of TurboID, RAG1, RAG2, and TurboID-fused RAG1 or RAG2 expression. Upper panel: samples probed with anti-HA antibody. Lower panel: samples probed with anti-RAG2 antibody. Black arrows indicate TurboID-RAG2 and RAG2. (D) In vivo recombination assay testing the activity of TurboID-fused RAG1 or RAG2. The assay was performed in Expi293F cells using a GFP reporter substrate. Both TurboID-RAG1 and TurboID-RAG2 exhibit recombination activity. (E) Western blot analysis of biotin labelling efficiency by TurboID alone or TurboID fused to RAG1 or RAG2, expressed in complex with their respective RAG-binding partners. Biotinylated proteins were detected using streptavidin-HRP.

TurboID is a highly efficient engineered biotin ligase with significantly enhanced catalytic activity, at least an order of magnitude higher than that of BirM. It enables rapid and efficient proximity-dependent biotinylation of nearby proteins in living cells [31]. To achieve broader and more representative coverage of the RAG interactome, we constructed expression vectors encoding full-length human RAG1 or RAG2 with TurboID fused at their N-termini ( Figure 2 A).

HEK293T cells were transiently transfected with either TurboID-RAG1 (co-expressing untagged RAG2) or TurboID-RAG2 (co-expressing untagged RAG1) to allow independent profiling of RAG1- and RAG2-proximal interactors in the RAG complex ( Figure 2B). Western blot analysis confirmed stable expression of both fusion proteins, and *in vivo* recombination assays demonstrated that TurboID fusion retained the catalytic activity of the RAG complex ( Figure 2C,D and Supplementary Figure S2). Remarkably, a brief 15-min biotin pulse was sufficient to achieve robust labelling of RAG-associated proteins from whole-cell lysates, underscoring the superior enzymatic efficiency of TurboID ( Figure 2E). Moreover, distinct banding patterns observed in streptavidin blotting suggested divergent interaction profiles between the two subunits ( Figure 2E). These observations highlight the impact of tag positioning and subunit context on proximity labelling outcomes of RAG1 vs RAG2 and reinforce the value of a spatially differentiated fusion strategy for dissecting subunit-specific interactomes within multiprotein complexes.

### Identification of RAG1- and RAG2-associated proteins

**Figure 3:**
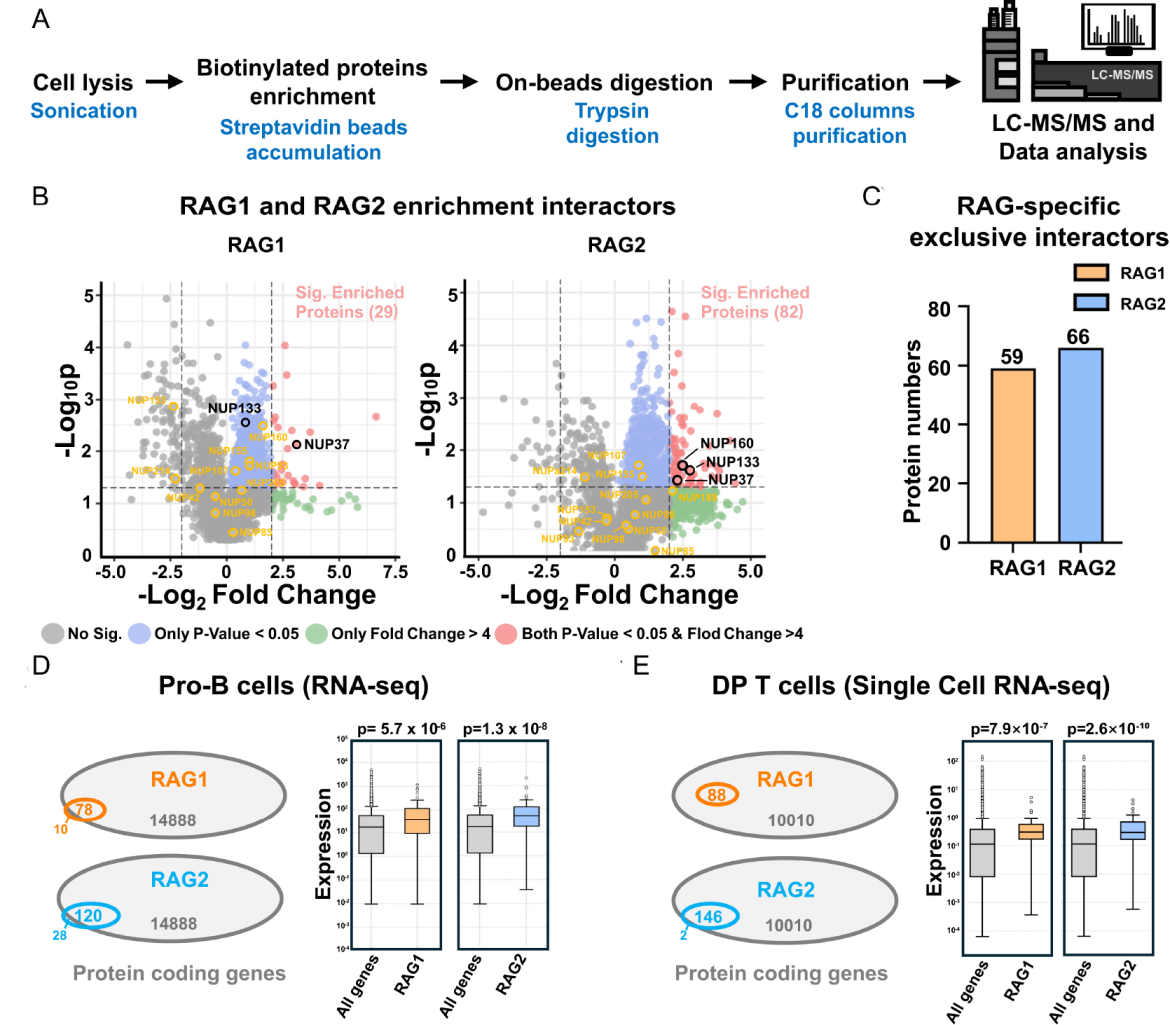
Figure 3 Identification of RAG1- and RAG2-interacting proteins (A) Schematic overview of the experimental workflow for the identification of RAG1/2 interactomes. Constructs expressing TurboID alone or TurboID fused to either RAG1 or RAG2 in complex with their respective binding partner were transfected into HEK293T cells. Following biotin incubation, cells were lysed, and biotinylated proteins were enriched using streptavidin beads. The captured proteins were subjected to on-bead digestion, followed by label-free quantitative proteomics using LC-MS/MS to identify proximity-labelled proteins. (B) Volcano plots showing biotinylated proteins enriched in TurboID-RAG1&RAG2 (left) and RAG1&TurboID-RAG2 (right) samples. Each protein is plotted based on its fold change (RAG/TurboID) and the two-tailed t test P value. Proteins with a fold change > 4 and P value < 0.05 are considered significantly enriched (highlighted in pink). Proteins selected for pull-down validation are marked with black circles; these and their interacting partners were analyzed using the STRING database, with those interactors also detected by mass spectrometry highlighted in yellow circles. RAG1 and RAG2 were enriched in 29 and 82 significantly differentially expressed proteins, respectively. (C) RAG-specific interacting proteins. Fifty-nine proteins were exclusively identified in RAG1 samples and 66 in RAG2 samples, with no detection in the TurboID control, highlighting their specific association with RAG1 or RAG2. KPNA3 and VPRBP are the proteins in the RAG1 interactome that were further investigated in this study. (D) Left: Venn diagram showing the intersection of proteins identified from RAG1 and RAG2 interactomes in this study with published pro-B-cell RNA-seq data. Right: Comparative expression analysis of RAG1 and RAG2 interactome proteins in pro-B-cell RNA-seq data. Proteins identified by TbID in HEK293 cells show significantly higher expression than the transcriptome-wide average in B cells. (E) Same analysis as in panel (D), performed using single-cell T-cell RNA-seq datasets. Proteins from the RAG1 and RAG2 interactomes show higher expression relative to the transcriptome-wide baseline in T cells.

Biotinylated proteins were enriched using streptavidin magnetic beads, extensively washed, and digested on-bead with trypsin as described previously [42]. The resulting peptides were analyzed by LC-MS/MS ( Figure 3A). From three independent biological replicates, we identified 29 significantly enriched proteins in the TurboID-RAG1 samples and 82 in the TurboID-RAG2 samples ( Figure 3B). An additional 59 proteins in the RAG1 group and 66 in the RAG2 group were detected exclusively in TurboID-tagged RAG samples but not in controls ( Figure 3C). Given that RAG is typically expressed in B and T lymphocytes, we compared the RAG1- and RAG2-enriched interactomes with transcriptome profiles of both human B and T cells [44]. All identified interactome proteins were expressed in B cells, suggesting that the interactome is relevant in a physiological context. To further assess their significance in T cells, we examined a published single-cell RNA-seq atlas of human thymic T-cell development [45], focusing on the double-positive (DP) thymocyte stage, which is characterized by peak RAG expression. Notably, all 88 RAG1-associated proteins and 146 of 148 RAG2-associated proteins were confirmed to be expressed in the relevant primary lymphocyte populations ( Figure 3D,E). In both progenitor (pro) B cells and double-positive (DP) T cells, the expression levels of proteins identified in the RAG1 or RAG2 interactomes were significantly higher than the average transcriptional level across all thymocyte-expressed genes. We further analyzed the expression levels of these identified interacting proteins in RAG-expressing immune cells, namely, pro-B cells and DN/DP-T cells, relative to other B and T lineage populations and selected native immune cell types. The results revealed a pronounced stronger enrichment of RAG interactors in pro-B and DN/DP-T cells ( Supplementary Figures S3 and S4). This finding reinforces the biological relevance of the identified interactomes across both major RAG-expressing lymphocyte lineages.

**Figure 4:**
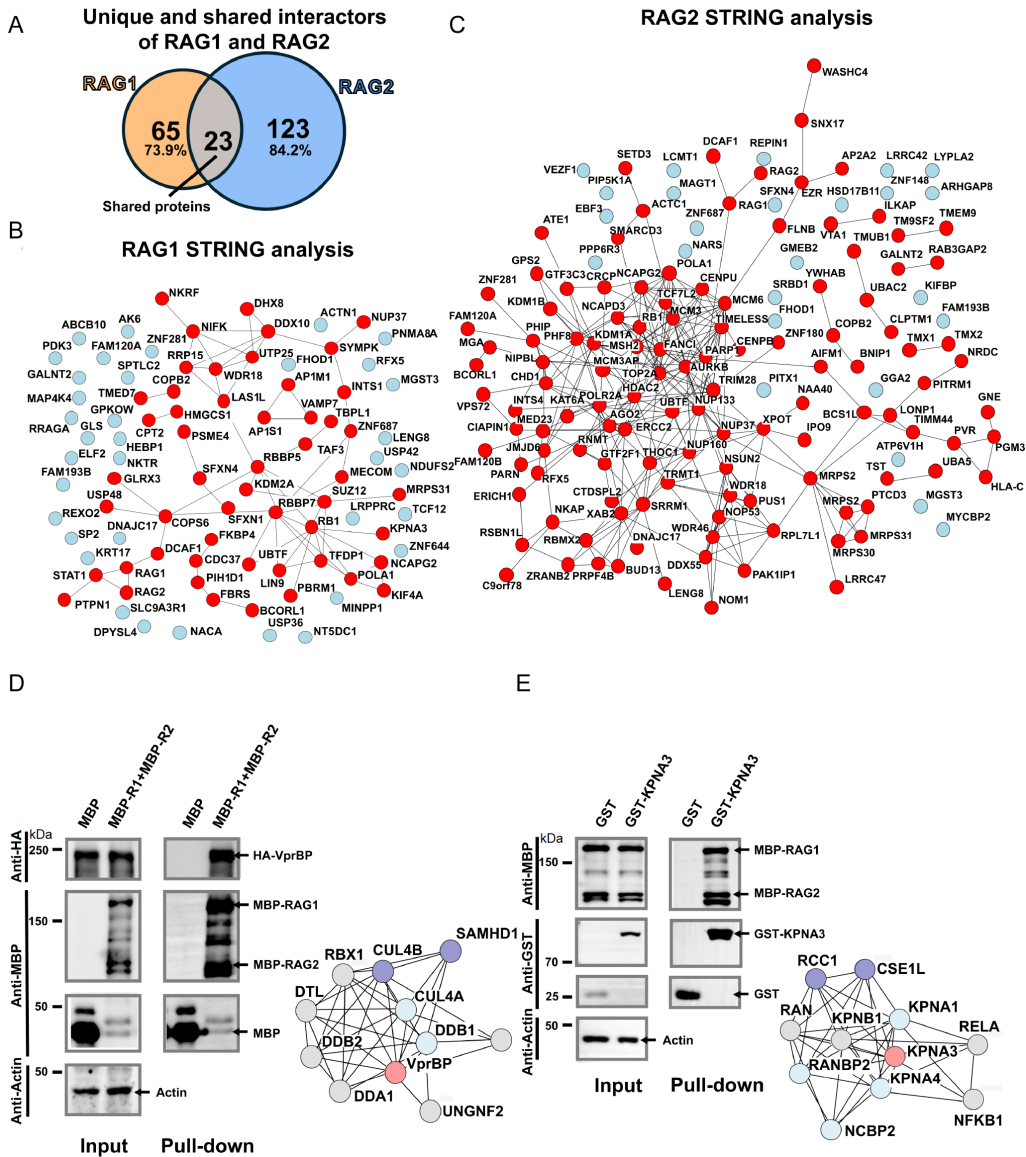
Figure 4 RAG1 and RAG2 exhibit distinct protein enrichment profiles (A) Venn diagram showing the overlap of proteins enriched by RAG1 and RAG2. Only 23 proteins (< 30%) are shared between the two subunits, highlighting their largely distinct interaction profiles. (B,C) STRING network analysis of proteins identified for RAG1 (B) and RAG2 (C). Red nodes represent proteins with interconnections, while blue nodes indicate proteins that lack functional associations with other identified proteins in the STRING database. Black lines indicate protein-protein associations that include experimentally validated and curated (known) interactions, computationally predicted interactions, and associations inferred from protein homology and co-expression patterns. (D) Left: GST pull-down assay in HEK293T cells verifying the interaction between MBP-fused RAG1/2 and HA-tagged VprBP. Right: STRING network analysis of VprBP. Red nodes represent VprBP, blue-violet nodes indicate proteins exhibiting significant expression differences compared to the control (mass spectrometry data), light blue nodes denote proteins without significant differences, and gray nodes represent proteins not detected. (E) Left: GST pull-down assay in HEK293T cells verifying the interaction between MBP-fused RAG1/2 and GST-tagged KPNA3. Right: the same STRING network analysis of KPNA3 as in (D).

Comparison of RAG1 and RAG2 interactomes revealed that only 23 proteins were common to both datasets—representing ~26.1% of the RAG1 interactome and ~15.8% of the RAG2 interactome ( Figure 4A). This limited overlap indicates that RAG1 and RAG2 engage in largely distinct sets of protein partners, underscoring the formation of subunit-specific regulatory clusters and reinforcing the view that RAG1 and RAG2 play spatially and functionally distinct roles within the RAG complex.

To further characterize the RAG1 and RAG2 interactomes, we performed STRING network analysis using all significantly enriched proteins—88 for RAG1 and 146 for RAG2 ( Figure 4B,C and Supplementary Table S3). Both RAG1 and RAG2 showed distinct interaction profiles, with substantial subsets of their associated proteins clustering into functionally related groups. This suggests that the identified interactors are not randomly distributed but are instead enriched in a series of related biological pathways. Indeed, this pattern was further underscored by the results of UniProt keyword enrichment analyses ( Supplementary Figure S5). Consistent with previous reports, we found that RAG is closely associated with ubiquitination-related functions, which aligns with prior findings that RAG interacts with VprBP (an E3 ubiquitin ligase receptor) [ 26, 46]. Notably, VprBP-related proteins were also identified among the RAG1-interacting proteins in our study ( Figure 4D). In addition, earlier reports demonstrated that KPNA1 can serve as a substrate for RAG1’s E3 ligase activity and undergo ubiquitination [47]. Although the absence of KPNA1 and KPNA2—importins previously shown to mediate RAG nuclear import [48]—our analysis identified KPNA3, a close homologue, along with several of its associated proteins ( Figure 4E). Moreover, *in vivo* GST pull-down assays demonstrated robust interactions between the hRAG proteins and both VprBP and KPNA3 ( Figure 4D,E). To further validate the specificity of RAG1- and RAG2-associated interactors, we selected KPNA3 and NAA40 as representative candidates and examined their interactions with RAG1 and RAG2 using *in vivo* pull-down assays. In the proteomic analysis, KPNA3 was specifically enriched in the RAG1 interactome, whereas NAA40 was specifically enriched in the RAG2 interactome, and both proteins exhibited clear subunit selectivity. In the *in vivo* GST pull-down assays, GST-KPNA3 specifically co-precipitated MBP-RAG1 but not MBP-RAG2; conversely, GST-NAA40 co-precipitated MBP-RAG2 but not MBP-RAG1. These results demonstrate the strong selectivity of the identified interactors for their respective subunits, providing experimental evidence that RAG1 and RAG2 engage distinct and at least partial non-overlapping interaction networks in cells ( Supplementary Figure S6).

### Functional divergence of RAG1 and RAG2 interactomes

**Figure 5:**
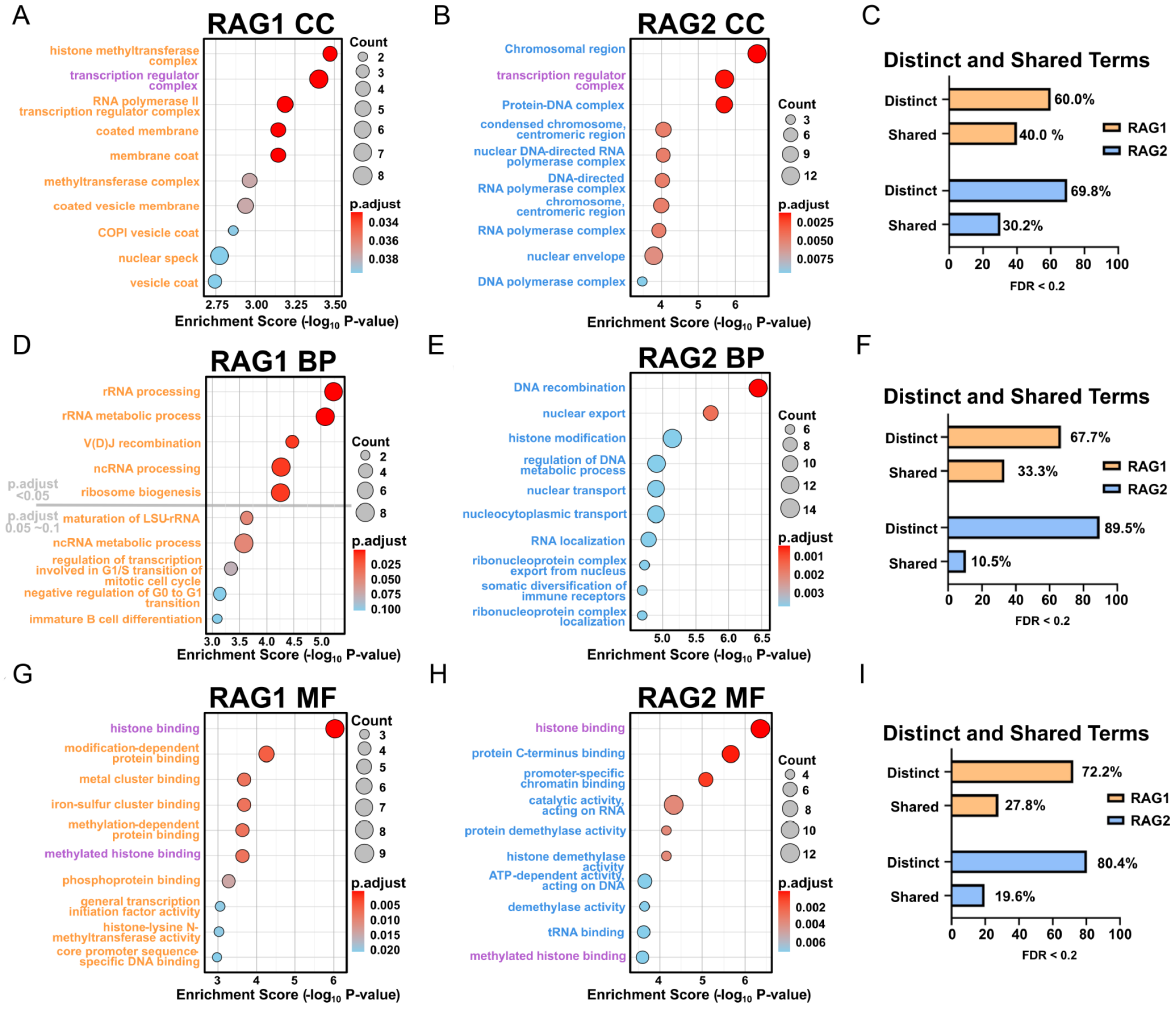
Figure 5 GO analysis of proteins identified in RAG1 and RAG2 proximity labelling (A,B) Cellular component (CC) analysis and comparison of RAG1- and RAG2-enriched proteins. The top 10 enriched terms are displayed. Terms specifically enriched by RAG1 and RAG2 are shown in orange and blue, respectively, while shared terms are shown in purple. (C) Comparison of significantly enriched CC terms between RAG1 and RAG2. GO terms with FDR-adjusted P values < 0.2 in either dataset were analyzed and categorized into two groups: Distinct, terms enriched exclusively by either RAG1 or RAG2; Shared, terms enriched in both datasets. (D,E) Biological process (BP) analysis and comparison of RAG1- and RAG2-enriched proteins. (F) Comparison of RAG1- and RAG2-enriched biological process (BP) terms, grouped into distinct and shared categories as defined in (C). (G,H) Molecular function (MF) analysis and comparison of RAG1- and RAG2-enriched proteins. (I) Comparison of RAG1- and RAG2-enriched molecular function (MF) terms, following the same classification criteria as in (C). In (D), only five terms had P.adjust < 0.05; therefore, five additional terms with P.adjust values below 0.2 were included in the list.

To delineate the functional differences in detail between RAG1 and RAG2 interactomes, we performed Gene Ontology (GO) enrichment analyses across cellular component (CC), biological process (BP), and molecular function (MF) categories. In CC, RAG1- and RAG2-associated proteins shared only one top term—“transcription regulator complex”—indicating the potential involvement of both subunits in transcriptional regulation ( Figure 5A,B). RAG1 interactors were enriched for transcriptional regulators and membrane trafficking components ( *e*. *g*., histone methyltransferase complex, RNA polymerase II complex, COPI vesicle coat), whereas RAG2 interactors were linked predominantly to chromatin structure, transcriptional machinery, and nuclear functions ( *e*. *g*., condensed chromosome, DNA polymerase complex) ( Figure 5A,B). Relaxing the threshold ( *P*.adjust < 0.2) modestly increased overlap (to ~40% for RAG1 and ~30% for RAG2) ( Figure 5C and Supplementary Table S4). However, many terms remained unique, highlighting distinct spatial and functional specialization.

For biological process (BP) terms, RAG1-associated proteins showed enrichment for rRNA-related processes and G1/S cell cycle regulators ( *e*. *g*., POLA1, TFDP1, and RB1), consistent with the restriction of RAG activity to the G1 phase ( Figure 5D). In contrast, RAG2 interactors were enriched for processes related to DNA recombination and repair, nucleocytoplasmic transport, and histone modification ( Figure 5E). Even at relaxed thresholds, overlap remained very limited (to ~33% for RAG1 and ~11% for RAG2), underscoring their functional divergence ( Figure 5F and Supplementary Table S4).

For Molecular Function (MF), both interactomes included histone-binding proteins but with distinct profiles: RAG1 interactors were enriched for activities such as lysine N-methyltransferase activity and modification-dependent protein binding, implicating histone methylation and broader post-translational regulation ( Figure 5G). RAG2 showed enrichment for demethylase activity and promoter-specific chromatin binding, suggesting a role in chromatin remodeling and transcriptional specificity ( Figure 5H). Once again, the overlap in MF terms was low (only ~20%–30% shared) even after relaxing the threshold ( Figure 5I and Supplementary Table S4).

Together, these findings reveal that although both RAG1 and RAG2 are involved in chromatin and epigenetic regulation, RAG1 is more strongly connected to cell cycle control and protein modification pathways, whereas RAG2 preferentially associates with chromatin dynamics, transcriptional specificity, and nuclear trafficking. This divergence indicates distinct regulatory functions beyond their shared catalytic role in V(D)J recombination.

### Interaction of nuclear transporter protein and nuclear pore complex with RAG

**Figure 6:**
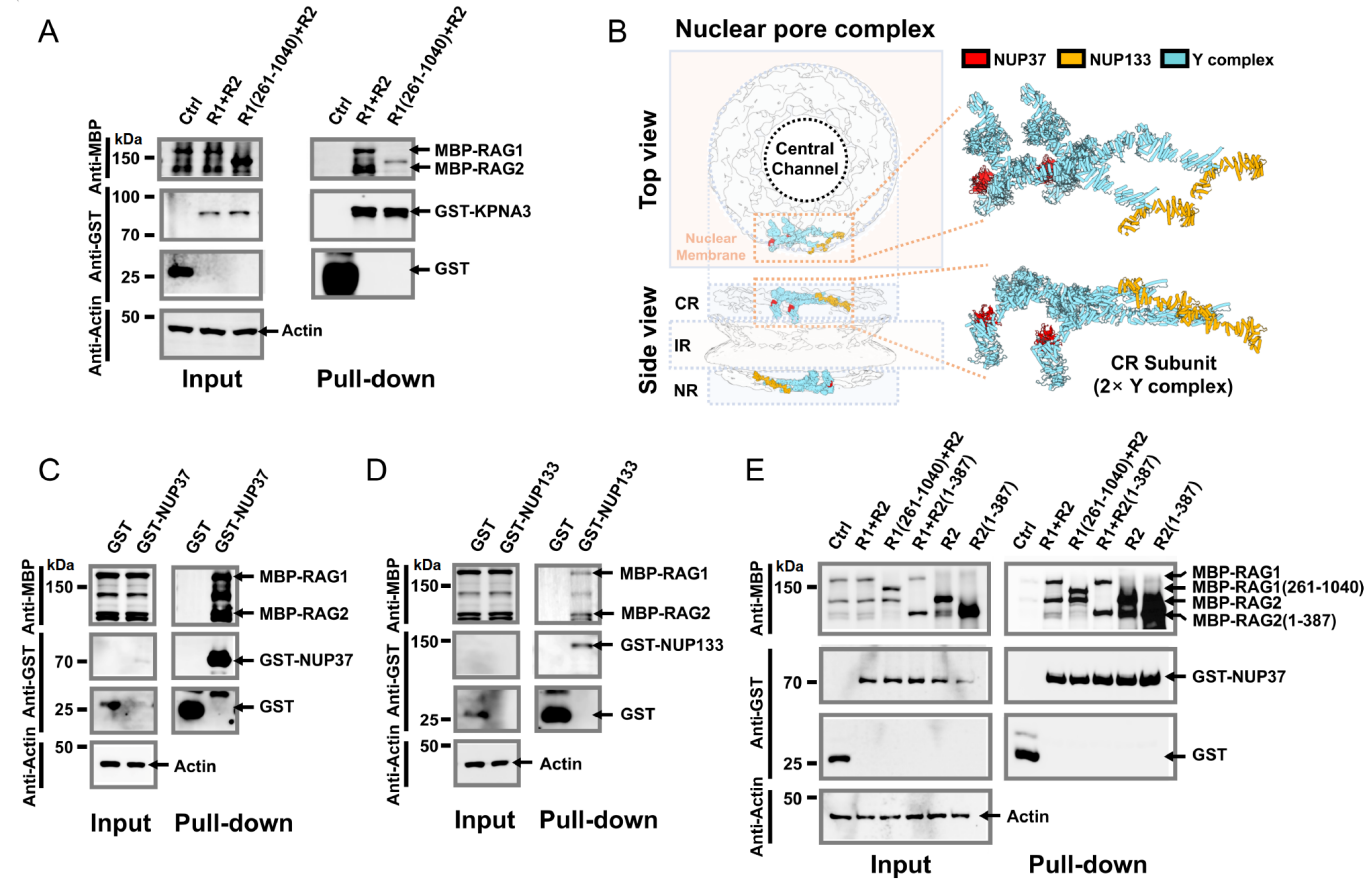
Figure 6 Interaction between RAG and nuclear transporter proteins and NPC (A) GST pull-down assay in HEK293T cells verifying the interaction between MBP-fused full-length RAG1/RAG2 or RAG1 261–1040/RAG2 and GST-tagged KPNA3. Deletion of RAG1 1–261 significantly reduced the association between RAG and KPNA3. (B) Schematic of the nuclear pore complex (NPC) and structure of the Y complex. Left: Top and side views of the NPC, with the background showing the cryo-ET structure (EMDB: EMD-12814); a single 2× Y complex is displayed as a surface model (PDB: [7PEQ]). Right: Enlarged top and side views of the 2×Y complex shown as a ribbon model. NUP37 and NUP133 are highlighted in red and yellow, respectively. (C,D) GST pull-down assay in HEK293T cells verifying the interaction between MBP-fused RAG1/RAG2 and GST-tagged NUP37 (C) or NUP133 (D). (E) GST pull-down assay in HEK293T cells to identify the minimal region of the RAG1/2 complex required for interaction with NUP37. The results show that the RAG2 core (residues 1–387) is sufficient for robust interaction with NUP37.

Nuclear import and export are critical for the physiological functions of RAG [49]. Both RAG1 and RAG2 contain nuclear localization signals (NLSs) that mediate their nucleocytoplasmic trafficking via karyopherin nuclear transport factors [ 50– 52]. In our study, we identified KPNA3 as a new RAG-interacting factor. Notably, the interaction between KPNA3 and RAG1 was markedly reduced when the RAG1 NLS was deleted (in the RAG1Δ1–261 mutant) ( Figure 6A), supporting the notion that KPNA3 recognizes RAG1 through its N-terminal NLS sequences. Additionally, our proteomic data also revealed several Y-complex subunits of the nuclear pore complex (NPC) ( Figure 3B), including NUP37 and NUP133, among the RAG2 interactors ( Supplementary Table S3). However, structural studies indicate that these proteins localize to the outer regions of the NPC cytoplasmic ring (CR) and nucleoplasmic ring (NR) instead of on the side of the nuclear pore channel [ 53– 56] ( Figure 6B), ruling out non-specific labelling due to RAG passage through the pore. *In vivo* pull-down assays confirmed robust interactions between NUP37 and RAG2, a weaker but detectable interaction between NUP133 and RAG2 ( Figure 6C,D). Furthermore, RAG2 lacking its putative NLS (RAG2 1–387) still interacted with NUP37 ( Figure 6E), suggesting that RAG2 engages the NPC through a previously unrecognized binding mode and may play yet-to-be-discovered roles in RAG nuclear transport.

## Discussion

In this study, we employed TurboID-based proximity labelling to systematically map the interaction networks of human RAG1 and RAG2, enabling spatially resolved characterization of the protein environments surrounding each subunit. Although the experiments were conducted in 293T cells rather than immune cells, we found that the proteins enriched in our study were expressed at higher levels in RAG-expressing immune populations (pro-B cells and DP-T cells) than in other immune cell types. This observation strengthens the physiological relevance of our findings and suggests that the identified interaction networks likely reflect the processes occurring in immune cells under physiological conditions. Accordingly, we declare that this work provides, for the first time at least in part, a reliable interactome map of human RAG proteins. Together, these data establish a robust framework for understanding the spatial organization of RAG1 and RAG2 interactions and substantially advance current knowledge of the human RAG protein interactome.

Despite forming a stable heterotetramer, RAG1 and RAG2 exhibit highly divergent interactomes. This divergence supports the idea that the two subunits engage in distinct cellular functions either independent of or parallel to their shared role in V(D)J recombination. Differences in spatial localization and evolutionary origin may underlie this division of function: RAG1 is known to have evolved from a Transib transposase, probably retaining both nuclease activity and putative regulatory capabilities [ 7 , 10, 57]. In contrast, RAG2 lacks a clearly defined evolutionary precursor, with hypotheses suggesting that it may have arisen independently as a regulatory factor that later co-evolved with RAG1. The striking asymmetry in their interactomes may thus reflect evolutionary specialization, positioning RAG1 and RAG2 as non-redundant functional modules within a broader regulatory framework.

One of the most striking findings was the enrichment of nuclear transport factors binding to the RAG2 core. It remains unclear whether the RAG complex translocates as a pre-assembled unit or whether RAG1 and RAG2 can enter or exit the nucleus independently. At present, it is also unclear whether RAG2’s interaction with NUP37 affects its nuclear trafficking. Resolving these dynamics will deepen our understanding of the subcellular regulation of RAG function and may also uncover extranuclear roles for RAG2. In contrast, RAG1-associated proteins were enriched in factors involved in cell cycle regulation. The restriction of RAG activity to the G1 phase is a critical mechanism for preserving genome integrity, and the identification of multiple G1/S checkpoint regulators in the RAG1 interactome suggests that RAG1 may interface directly with the machinery controlling this restriction.

In addition to the proteins consistently enriched in our datasets, we also examined factors previously implicated in RAG function, such as HMGB1. Although HMGB1 is widely regarded as a RAG cofactor, in our study, it displayed only modest enrichment (1.78-fold) and did not reach statistical significance. This outcome is consistent with structural studies demonstrating that HMGB1 does not directly interact with RAG but instead binds to the substrate RSS DNA to facilitate RAG/RSS complex formation [58]. Moreover, given that immunoglobulin and TCR loci are not fully accessible in 293T cells, the frequency of RAG binding to its substrate DNA—and consequently the probability of forming indirect complexes with HMGB1—is likely reduced. These observations support the interpretation that the majority of proteins identified in the RAG1/2 interactomes represent direct physical interactors rather than indirect cofactors.

By mapping the RAG complex interactome at the subunit level, we provide new evidence that human RAG1 and RAG2 may play specialized physiological roles beyond their canonical biochemical function in V(D)J recombination. However, it remains unknown whether the two subunit-specific interaction networks operate cooperatively, redundantly, or interdependently or how they influence lymphocyte development and immune homeostasis. Furthermore, although RAG1 and RAG2 typically function together as tetrameric complexes, some studies suggest that free (unbound) forms of RAG1 or RAG2 may also exist in cells [ 34, 35]. Whether such forms possess independent biological activity or act as regulatory reserves is an open question that warrants direct experimental investigation.

## Supporting information

25764Supplementary_data

## Supplementary Data

Supplementary data are available at *Acta Biochimica et Biophysica Sinica* online.

## References

[1] Nishana M, Raghavan SC (2012). Role of recombination activating genes in the generation of antigen receptor diversity and beyond. Immunology.

[2] Schatz DG (2004). Antigen receptor genes and the evolution of a recombinase. Semin Immunol.

[3] Schatz DG, Swanson PC (2011). V(D)J recombination: mechanisms of initiation. Annu Rev Genet.

[4] Schatz DG, Ji Y (2011). Recombination centres and the orchestration of V(D)J recombination. Nat Rev Immunol.

[5] Gellert M (2002). V(D)J recombination: RAG proteins, repair factors, and regulation. Annu Rev Biochem.

[6] Agrawal A, Eastman QM, Schatz DG (1998). Transposition mediated by RAG1 and RAG2 and its implications for the evolution of the immune system. Nature.

[7] Carmona LM, Schatz DG (2017). New insights into the evolutionary origins of the recombination-activating gene proteins and V(D)J recombination. FEBS J.

[8] Hiom K, Melek M, Gellert M (1998). DNA transposition by the RAG1 and RAG2 proteins. Cell.

[9] Huang S, Tao X, Yuan S, Zhang Y, Li P, Beilinson HA, Zhang Y (2016). Discovery of an active RAG transposon illuminates the origins of V(D)J recombination. Cell.

[10] Kapitonov VV, Jurka J, Nemazee D (2005). RAG1 core and V(D)J recombination signal sequences were derived from transib transposons. PLoS Biol.

[11] Liu C, Zhang Y, Liu CC, Schatz DG (2022). Structural insights into the evolution of the RAG recombinase. Nat Rev Immunol.

[12] Martin EC, Vicari C, Tsakou-Ngouafo L, Pontarotti P, Petrescu AJ, Schatz DG (2020). Identification of RAG-like transposons in protostomes suggests their ancient bilaterian origin. Mobile DNA.

[13] Zhang Y, Cheng TC, Huang G, Lu Q, Surleac MD, Mandell JD, Pontarotti P (2019). Transposon molecular domestication and the evolution of the RAG recombinase. Nature.

[14] Zhang Y, Corbett E, Wu S, Schatz DG (2020). Structural basis for the activation and suppression of transposition during evolution of the RAG recombinase. EMBO J.

[15] Chi X, Li Y, Qiu X (2020). V(D)J recombination, somatic hypermutation and class switch recombination of immunoglobulins: mechanism and regulation. Immunology.

[16] Teng G, Schatz DG. Regulation and evolution of the RAG recombinase.
Adv Immunol 2015, 128: 1-39. https://doi.org/10.1016/bs.ai.2015.07.002.

[17] Liu Y, Subrahmanyam R, Chakraborty T, Sen R, Desiderio S (2007). A plant homeodomain in Rag-2 that binds hypermethylated lysine 4 of histone H3 is necessary for efficient antigen-receptor-gene rearrangement. Immunity.

[18] Maman Y, Teng G, Seth R, Kleinstein SH, Schatz DG (2016). RAG1 targeting in the genome is dominated by chromatin interactions mediated by the non-core regions of RAG1 and RAG2. Nucleic Acids Res.

[19] Matthews AGW, Kuo AJ, Ramón-Maiques S, Han S, Champagne KS, Ivanov D, Gallardo M (2007). RAG2 PHD finger couples histone H3 lysine 4 trimethylation with V(D)J recombination. Nature.

[20] Shimazaki N, Tsai AG, Lieber MR (2009). H3K4me3 stimulates the V(D)J RAG complex for both nicking and hairpinning in trans in addition to tethering in
*cis*: implications for translocations. Mol Cell.

[21] Deng Z, Liu H, Liu X (2015). RAG1-mediated ubiquitylation of histone H3 is required for chromosomal V(D)J recombination. Cell Res.

[22] Grazini U, Zanardi F, Citterio E, Casola S, Goding CR, McBlane F (2010). The RING domain of RAG1 ubiquitylates histone H3: a novel activity in chromatin-mediated regulation of V(D)J joining. Mol Cell.

[23] Singh SK, Gellert M (2015). Role of RAG1 autoubiquitination in V(D)J recombination. Proc Natl Acad Sci USA.

[24] Yurchenko V, Xue Z, Sadofsky M (2003). The RAG1 N-terminal domain is an E3 ubiquitin ligase. Genes Dev.

[25] Kassmeier MD, Mondal K, Palmer VL, Raval P, Kumar S, Perry GA, Anderson DK (2012). VprBP binds full-length RAG1 and is required for B-cell development and V(D)J recombination fidelity. EMBO J.

[26] Schabla NM, Perry GA, Palmer VL, Swanson PC (2018). VprBP (DCAF1) regulates RAG1 expression independently of dicer by mediating RAG1 degradation. J Immunol.

[27] Raval P, Kriatchko AN, Kumar S, Swanson PC (2008). Evidence for Ku70/Ku80 association with full-length RAG1. Nucleic Acids Res.

[28] Roux KJ, Kim DI, Burke B, May DG (2018). BioID: a screen for protein-protein interactions. CP Protein Sci.

[29] Roux KJ, Kim DI, Raida M, Burke B (2012). A promiscuous biotin ligase fusion protein identifies proximal and interacting proteins in mammalian cells. J Cell Biol.

[30] Brecht RM, Liu CC, Beilinson HA, Khitun A, Slavoff SA, Schatz DG (2020). Nucleolar localization of RAG1 modulates V(D)J recombination activity. Proc Natl Acad Sci USA.

[31] Branon TC, Bosch JA, Sanchez AD, Udeshi ND, Svinkina T, Carr SA, Feldman JL (2018). Efficient proximity labeling in living cells and organisms with TurboID. Nat Biotechnol.

[32] Cho KF, Branon TC, Udeshi ND, Myers SA, Carr SA, Ting AY (2020). Proximity labeling in mammalian cells with TurboID and split-TurboID. Nat Protoc.

[33] Kim MS, Lapkouski M, Yang W, Gellert M (2015). Crystal structure of the V(D)J recombinase RAG1-RAG2. Nature.

[34] Ji Y, Resch W, Corbett E, Yamane A, Casellas R, Schatz DG (2010). The
*in vivo* pattern of binding of RAG1 and RAG2 to antigen receptor loci. Cell.

[35] Zhang YH, Shetty K, Surleac MD, Petrescu AJ, Schatz DG (2015). Mapping and quantitation of the interaction between the recombination activating gene proteins RAG1 and RAG2. J Biol Chem.

[36] Yu G (2024). Thirteen years of clusterProfiler. Innovation.

[37] Yu G, Wang LG, Han Y, He QY (2012). clusterProfiler: an R package for comparing biological themes among gene clusters. OMICS.

[38] Wickham H. ggplot2: elegant graphics for data analysis New York.
NY: Springer 2009, 10. https://doi.org/10.1103/PhysRevE.83.036701.

[39] Grundy GJ, Ramón-Maiques S, Dimitriadis EK, Kotova S, Biertümpfel C, Heymann JB, Steven AC (2009). Initial stages of V(D)J recombination: the organization of RAG1/2 and RSS DNA in the postcleavage complex. Mol Cell.

[40] Ru H, Chambers MG, Fu TM, Tong AB, Liao M, Wu H (2015). Molecular mechanism of V(D)J recombination from synaptic RAG1-RAG2 complex structures. Cell.

[41] Kim DI, Kc B, Zhu W, Motamedchaboki K, Doye V, Roux KJ (2014). Probing nuclear pore complex architecture with proximity-dependent biotinylation. Proc Natl Acad Sci USA.

[42] Sears RM, May DG, Roux KJ. BioID as a tool for protein-proximity labeling in living cells.
Methods Mol Biol 2019, 2012: 299-313. https://doi.org/10.1007/978-1-4939-9546-2_15.

[43] May DG, Scott KL, Campos AR, Roux KJ (2020). Comparative application of BioID and TurboID for protein-proximity biotinylation. Cells.

[44] Ma Q, Caillier SJ, Muzic S, Wilson MR, Henry RG, Cree BAC, Hauser SL (2021). Specific hypomethylation programs underpin B cell activation in early multiple sclerosis. Proc Natl Acad Sci USA.

[45] Park JE, Botting RA, Domínguez Conde C, Popescu DM, Lavaert M, Kunz DJ, Goh I (2020). A cell atlas of human thymic development defines T cell repertoire formation. Science.

[46] Schabla NM, Swanson PC, Fugmann SD (2021). The CRL4VPRBP(DCAF1) E3 ubiquitin ligase directs constitutive RAG1 degradation in a non-lymphoid cell line. PLoS One.

[47] Simkus C, Makiya M, Jones JM (2009). Karyopherin alpha 1 is a putative substrate of the RAG1 ubiquitin ligase. Mol Immunol.

[48] Chao J, Rothschild G, Basu U (2014). Ubiquitination events that regulate recombination of immunoglobulin loci gene segments. Front Immunol.

[49] Braams M, Pike-Overzet K, Staal FJT (2023). The recombinase activating genes: architects of immune diversity during lymphocyte development. Front Immunol.

[50] Arbuckle JL, Rahman NS, Zhao S, Rodgers W, Rodgers KK (2011). Elucidating the domain architecture and functions of non-core RAG1: the capacity of a non-core zinc-binding domain to function in nuclear import and nucleic acid binding. BMC Biochem.

[51] Ross AE, Vuica M, Desiderio S (2003). Overlapping signals for protein degradation and nuclear localization define a role for intrinsic RAG-2 nuclear uptake in dividing cells. Mol Cell Biol.

[52] Spanopoulou E, Cortes P, Shih C, Huang CM, Silver DP, Svec P, Baltimore D (1995). Localization, interaction, and RNA binding properties of the V(D)J recombination-activating proteins RAG1 and RAG2. Immunity.

[53] Asakawa H, Kojidani T, Yang HJ, Ohtsuki C, Osakada H, Matsuda A, Iwamoto M (2019). Asymmetrical localization of Nup107-160 subcomplex components within the nuclear pore complex in fission yeast. PLoS Genet.

[54] Bilokapic S, Schwartz TU (2012). Molecular basis for Nup37 and ELY5/ELYS recruitment to the nuclear pore complex. Proc Natl Acad Sci USA.

[55] Lin DH, Hoelz A (2019). The structure of the nuclear pore complex (an update). Annu Rev Biochem.

[56] Schuller AP, Wojtynek M, Mankus D, Tatli M, Kronenberg-Tenga R, Regmi SG, Dip PV (2021). The cellular environment shapes the nuclear pore complex architecture. Nature.

[57] Liu C, Yang Y, Schatz DG (2019). Structures of a RAG-like transposase during cut-and-paste transposition. Nature.

[58] Little AJ, Corbett E, Ortega F, Schatz DG (2013). Cooperative recruitment of HMGB1 during V(D)J recombination through interactions with RAG1 and DNA. Nucleic Acids Res.

