# Supplementary material for: Proximity-based proteomic profiling uncovers distinct interactome of human RAG1 and RAG2: RAG1&RAG2 interaction networks
Source: Acta Biochim Biophys Sin (Shanghai). 2026 Jan 27;58(4):756–67. doi: 10.3724/abbs.2025246 (PMC13107022; doi:10.3724/abbs.2025246)
Supplement: 25764Supplementary_data [file 25764Supplementary_data.docx]

**Supplementary Figure legends**


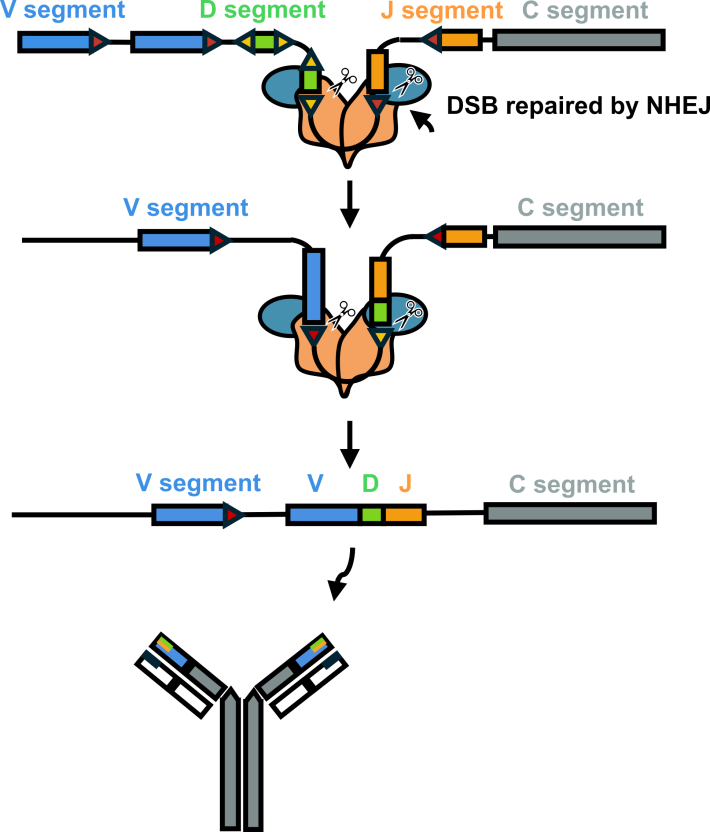


**Supplementary Figure S1. Schematic overview of V(D)J recombination mediated by the RAG recombinase** The RAG1-RAG2 complex specifically recognizes recombination signal sequences (RSS) flanking the variable (V), diversity (D), and joining (J) gene segments at immunoglobulin (Ig) or T-cell receptor (TCR) loci. Upon binding to a 12-RSS and a 23-RSS in a synaptic complex, RAG mediates site-specific cleavage at the junctions where coding sequences meet their corresponding recombination signal sequences (RSS), generating double-strand breaks (DSBs). Productive recombination is guided by the “12/23 rule,” which restricts recombination to occur only between gene segments flanked by RSSs of different spacer lengths—one with a 12 bp spacer and the other with a 23 bp spacer—thereby ensuring proper V(D)J joining. Subsequent processing by the non-homologous end joining (NHEJ) machinery facilitates ligation of coding ends to generate functional antigen receptor genes. This process enables the assembly of a diverse antigen receptor repertoire essential for adaptive immunity.


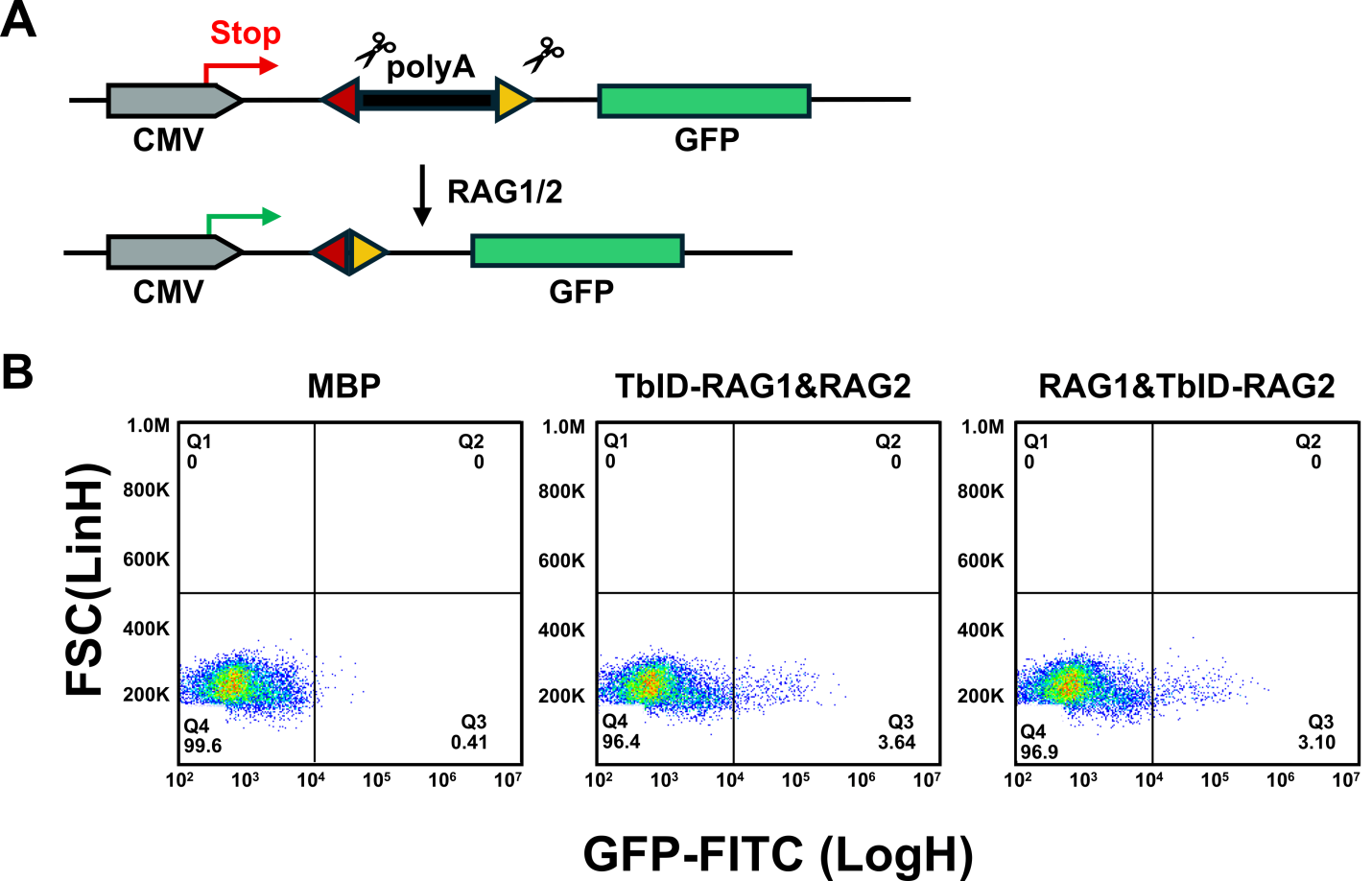


**Supplementary Figure S2. Schematic of GFP reporter system and FACS analysis of recombination activity** (A) Red and yellow triangles represent 12RSS and 23RSS, respectively. A transcriptional stop sequence containing a polyA signal is flanked by RSSs in convergent orientation and separates the GFP coding sequence from its upstream promoter. Upon RAG-mediated recombination and excision of the stop cassette, GFP is expressed as a readout of recombination activity. (B) In the FACS assay, cells were sequentially gated as “Intact cells → live cells (DAPI^−^),” and GFP^+^ populations were quantified in the Q3 quadrant. The control group (reporter only, left panel) showed a very low background with 0.41% GFP^+^ cells. In contrast, both experimental conditions (middle and right panels) exhibited clear GFP positivity, with 3.64% and 3.10% GFP^+^ cells, respectively, indicating that TbID-R1 and TbID-R2 both possess effective recombination activity.

**
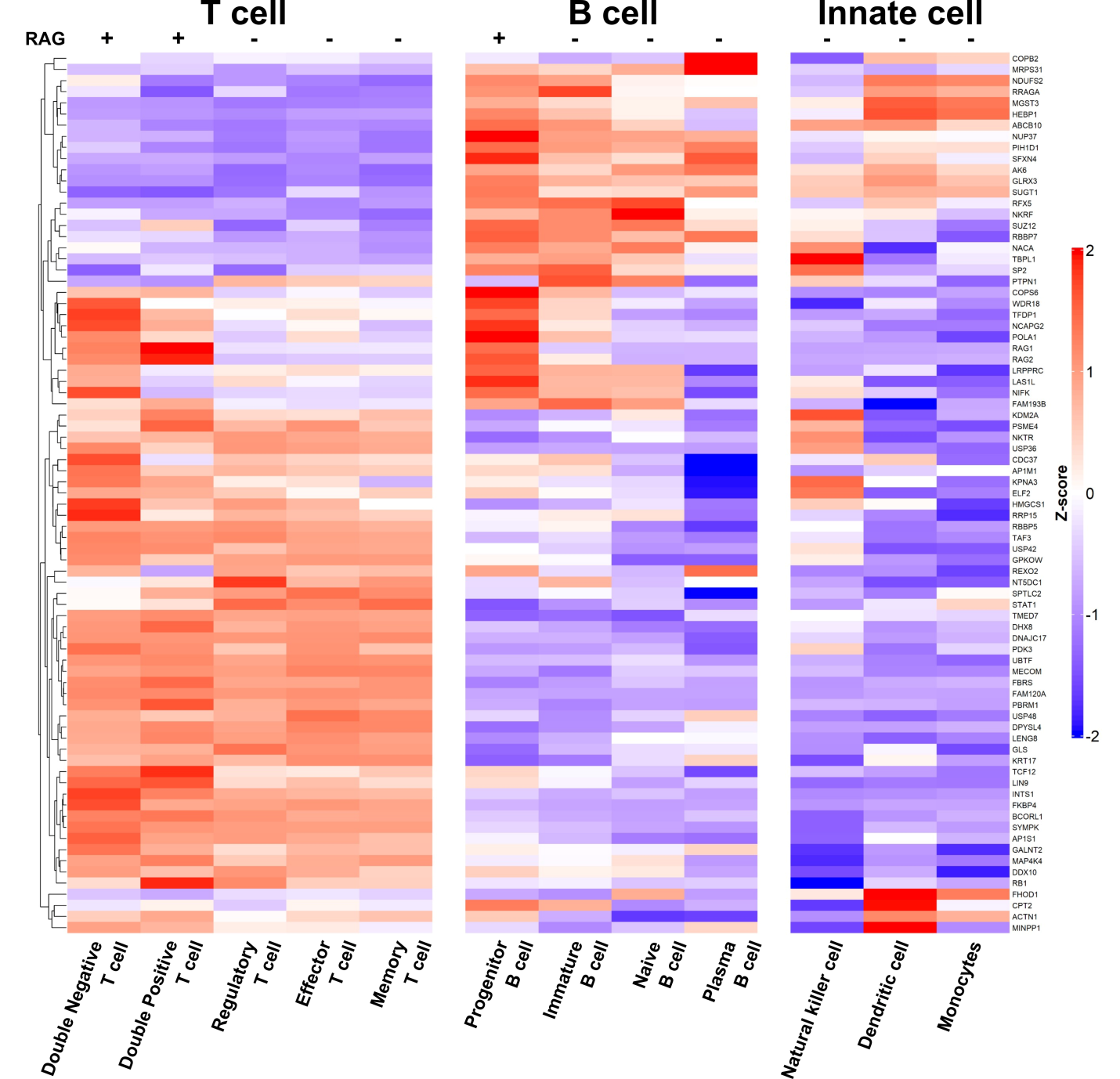
**

**Supplementary Figure S3. Normalized expression of RAG1 interactome genes across T, B and innate immune cells** Nine of the 88 RAG1 interactome genes were not present in the reference datasets and therefore are not shown in the heatmap. These genes include ZNF281, DCAF1, NHERF1, PNMA8A, UTP25, VAMP7, KIF4A, ZNF644, and ZNF687.

**
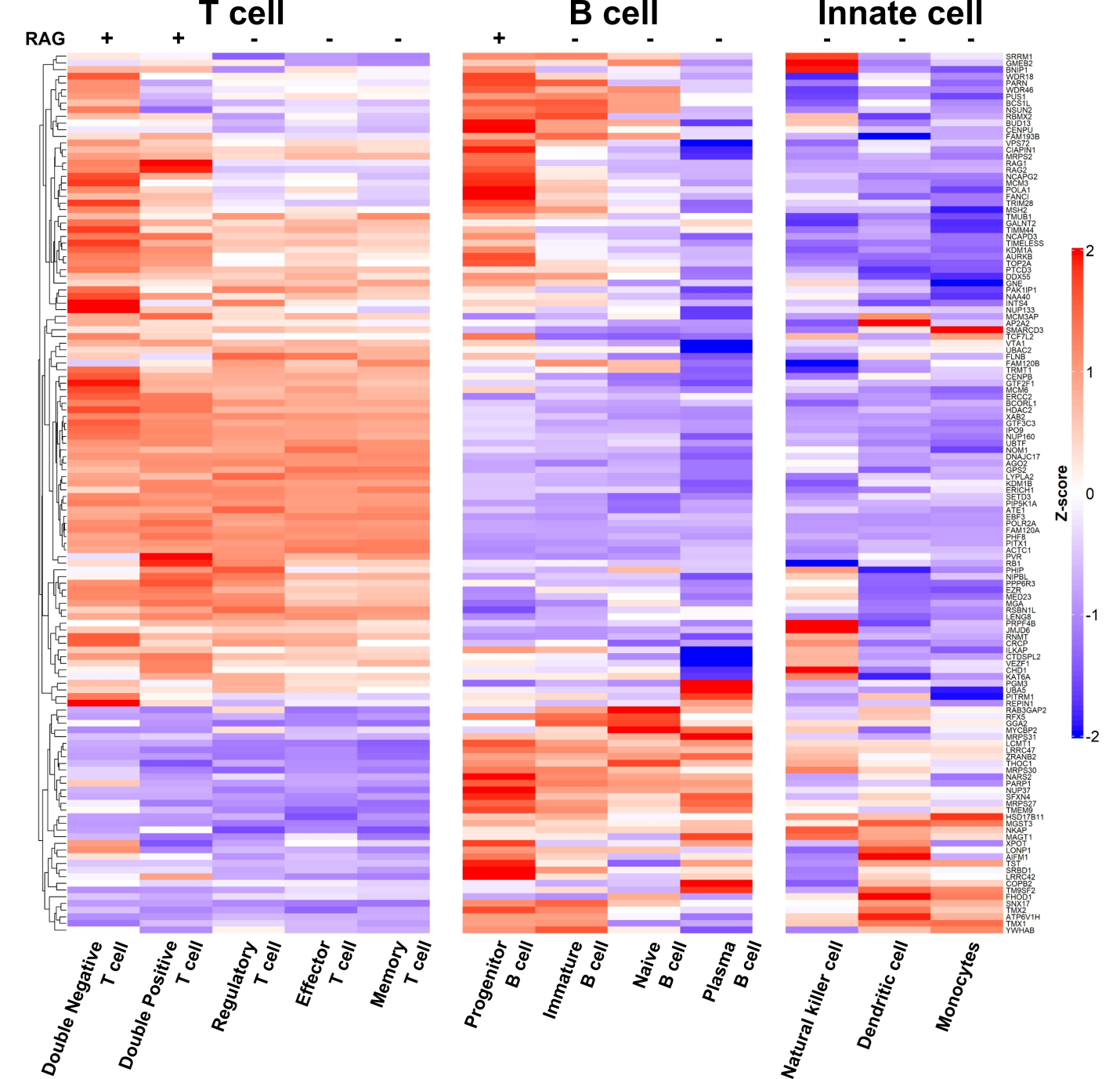
**

**Supplementary Figure S4. Normalized expression of RAG2 interactome genes across T, B and innate immune cells** Thirteen of the 146 RAG1 interactome genes were not present in the reference datasets and therefore are not shown in the heatmap. These genes include *ARHGAP8, C9ORF78, DCAF1, HLA-C, KIFBP, NOP53, NRDC, RPL7L1, WASHC4, ZNF148, ZNF180, ZNF281, and ZNF687*.


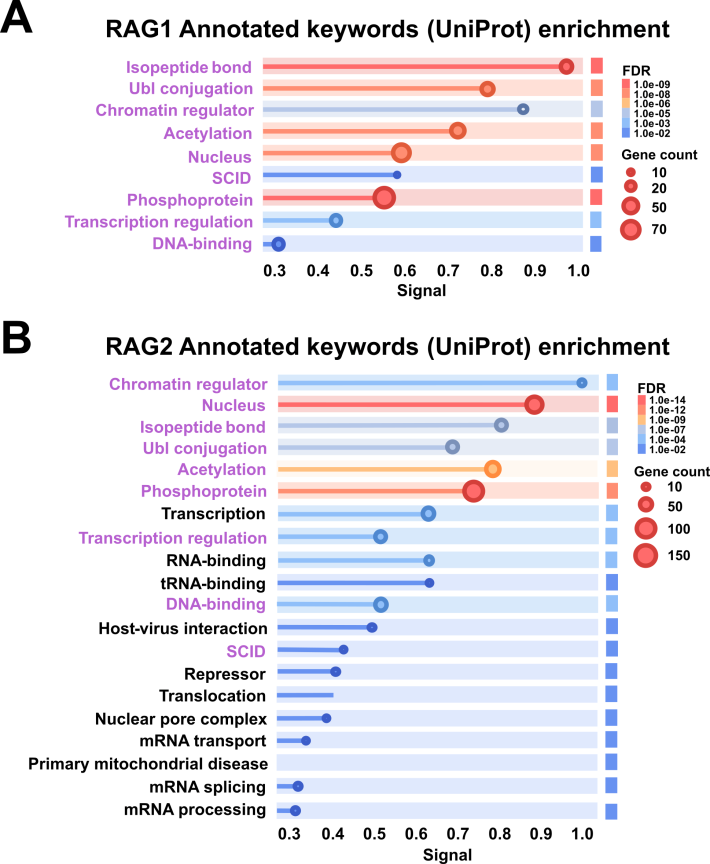


**Supplementary Figure S5. Annotated keyword enrichment (UniProt) of RAG1-associated (A) and RAG2-associated (B) proteins** The enriched keywords identified in RAG2 samples encompass all those enriched in RAG1 samples (highlighted in purple), indicating a broader interaction network for RAG2.

**
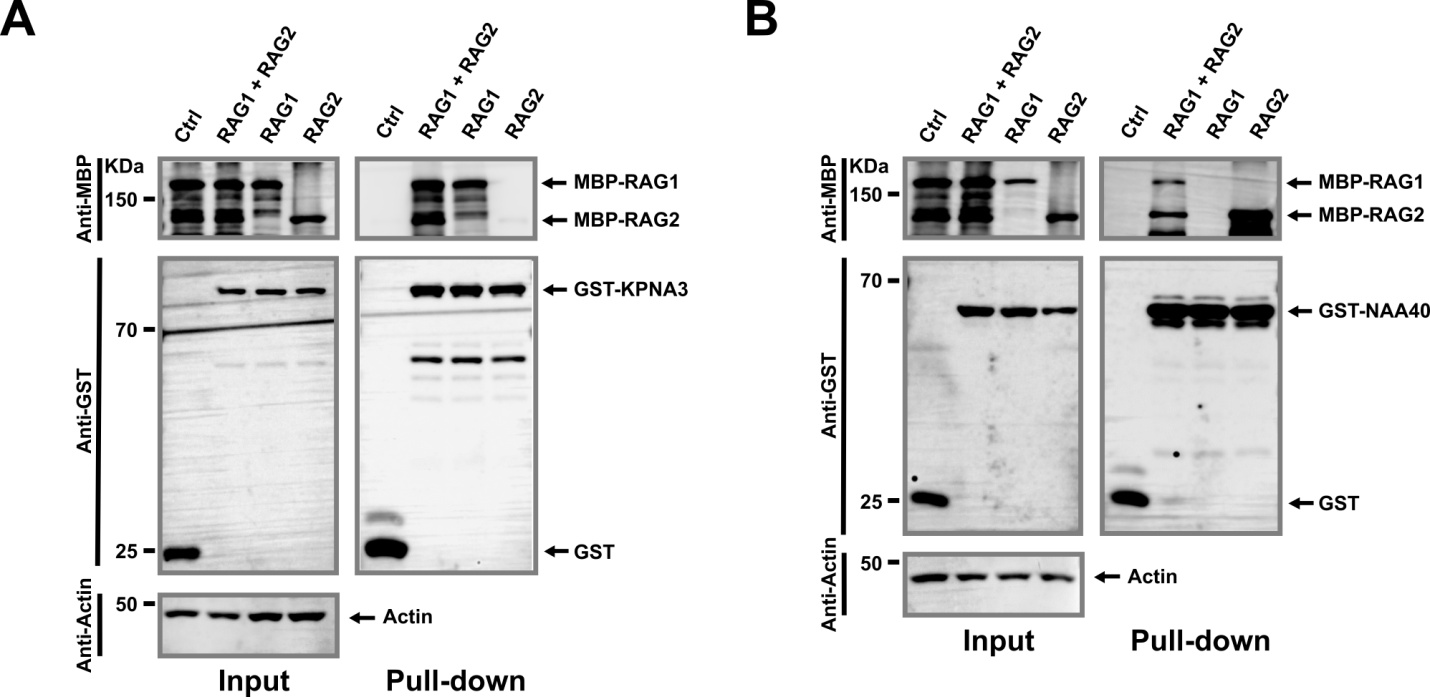
**

**Supplementary Figure S6. Subunit-selective interactions of KPNA3 and NAA40 with RAG1 or RAG2** (A) GST pull-down assay in HEK293T cells verifying the interaction between MBP-fused full-length RAG1/RAG2 and GST-tagged KPNA3. KPNA3 predominantly interacted with the RAG1/RAG2 complex or with RAG1 alone but showed no detectable interaction with RAG2 alone. (B) GST pull-down assay in HEK293T cells verifying the interaction between MBP-fused full-length RAG1/RAG2 and GST-tagged NAA40. NAA40 interacted with RAG2, but showed no detectable interaction with RAG1 or RAG1/2 complex.

**Supplementary Table S1. Constructs used in this study**

| Plasmid | Source | Identifier |
| --- | --- | --- |
| pTT5- HA-TbID | This study | N.A. |
| pTT5- HA-TbID-RAG1-IRES-RAG2 | This study | N.A. |
| pTT5- HA-RAG1-IRES- TbID-RAG2 | This study | N.A. |
| pEBB-HA-VprBP(DCAF1) | This study | N.A. |
| pTT5-MBP-RAG1-IRES-RAG2 | This study | N.A. |
| pTT5-MBP-RAG1-FL | This study | N.A. |
| pTT5-MBP-RAG1-Δ261 | This study | N.A. |
| pTT5-MBP-RAG2-FL | This study | N.A. |
| pTT5-MBP-RAG2-1-387 | This study | N.A. |
| pTT5-GST-KPNA3 | This study | N.A. |
| pTT5-GST-NUP37 | This study | N.A. |
| pTT5-GST-NUP133 | This study | N.A. |
| pTT5-GST-NAA40 | This study | N.A. |

**Supplementary Table S2. Antibody used in this study**

| Antibodies | Source | Identifier |
| --- | --- | --- |
| Rabbit monoclonal anti-HA | Cell Signaling Technology | Cat. #3724S |
| Anti-RAG2 | A gift from David Schatz | N.A. |
| Mouse monoclonal anti-MBP | Abmart | Cat. #M20051 |
| Mouse monoclonal anti-GST | Proteintech | Cat. #66001-2-Ig |
| Rabbit monoclonal anti-β-actin | Abmart | Cat. #P30002 |
| HRP-conjugated secondary antibodies (goat anti-rabbit) | Abmart | Cat. #M21001 |
| HRP-conjugated secondary antibodies (goat anti-mouse) | Abmart | Cat. #21002 |
| treptavidin-HRP conjugate | Sangon Biotech | Cat. #B110053 |

**Supplementary Table S3. Representative RAG-enriched or specific proteins**

**Supplementary Table S3A. RAG1-enriched or specific proteins**

| ACTN1 | GLRX3 | NUP37 |
| --- | --- | --- |
| AP1M1 | LAS1L | PTPN1 |
| AP1S1 | LRPPRC | RRP15 |
| CDC37 | MGST3 | SPTLC2 |
| COPB2 | MRPS31 | SYMPK |
| COPS6 | NACA | UBTF |
| CPT2 | NDUFS2 | USP36 |
| DDX10 | NIFK | WDR18 |
| FKBP4 | NKRF | ZNF281 |
| GALNT2 | NKTR |  |
| ABCB10 | KRT17 | RBBP7 |
| AK6 | LENG8 | REXO2 |
| BCORL1 | LIN9 | RFX5 |
| DCAF1 | RRAGA | DHX8 |
| MAP4K4 | SFXN4 | DNAJC17 |
| MECOM | SP2 | DPYSL4 |
| MINPP1 | STAT1 | ELF2 |
| NCAPG2 | SUGT1 | FAM120A |
| NHERF1 | SUZ12 | FAM193B |
| NT5DC1 | TAF3 | FBRS |
| PBRM1 | TBPL1 | FHOD1 |
| PDK3 | TCF12 | GLS |
| PIH1D1 | TFDP1 | GPKOW |
| PNMA8A | TMED7 | HEBP1 |
| POLA1 | USP42 | HMGCS1 |
| PSME4 | USP48 | INTS1 |
| RAG1 | UTP25 | KDM2A |
| RAG2 | VAMP7 | KIF4A |
| RB1 | ZNF644 | KPNA3 |
| RBBP5 | ZNF687 |  |

**Supplementary Table 3B. RAG2-enriched or specific proteins**

| ACTC1 | IPO9 | PRPF4B |
| --- | --- | --- |
| AIFM1 | KIFBP | PTCD3 |
| AP2A2 | LCMT1 | RNMT |
| AURKB | LONP1 | RPL7L1 |
| BCS1L | LRRC47 | SFXN4 |
| BNIP1 | LYPLA2 | SMARCD3 |
| BUD13 | MCM3 | SNX17 |
| C9orf78 | MCM6 | SRRM1 |
| CIAPIN1 | MGA | THOC1 |
| COPB2 | MGST3 | TIMELESS |
| CTDSPL2 | MRPS2 | TIMM44 |
| DCAF1 | MRPS27 | TM9SF2 |
| DDX55 | MRPS30 | TMUB1 |
| ERCC2 | MRPS31 | TMX1 |
| ERICH1 | MSH2 | TRIM28 |
| EZR | NARS2 | TST |
| FAM120A | NSUN2 | UBA5 |
| FANCI | NUP133 | UBTF |
| FLNB | NUP160 | VPS72 |
| GALNT2 | NUP37 | VTA1 |
| GNE | PAK1IP1 | WDR18 |
| GPS2 | PARP1 | XAB2 |
| GTF3C3 | PGM3 | XPOT |
| HDAC2 | PHF8 | YWHAB |
| HLA-C | PIP5K1A | ZNF148 |
| HSD17B11 | PITRM1 | ZNF281 |
| ILKAP | PPP6R3 | ZRANB2 |
| AGO2 | KDM1B | RAB3GAP2 |
| ARHGAP8 | LENG8 | RAG1 |
| ATE1 | LRRC42 | RAG2 |
| ATP6V1H | MAGT1 | RB1 |
| BCORL1 | MCM3AP | RBMX2 |
| CENPB | MED23 | REPIN1 |
| CENPU | MYCBP2 | RFX5 |
| CHD1 | NAA40 | RSBN1L |
| CLPTM1 | NCAPD3 | SETD3 |
| CRCP | NCAPG2 | SRBD1 |
| DNAJC17 | NIPBL | TCF7L2 |
| EBF3 | NKAP | TMEM9 |
| FAM120B | NOM1 | TMX2 |
| FAM193B | NOP53 | TOP2A |
| FHOD1 | NRDC | TRMT1 |
| GGA2 | PARN | UBAC2 |
| GMEB2 | PHIP | VEZF1 |
| GTF2F1 | PITX1 | WASHC4 |
| INTS4 | POLA1 | WDR46 |
| JMJD6 | POLR2A | ZNF180 |
| KAT6A | PUS1 | ZNF687 |
| KDM1A | PVR |  |

**Supplementary Table 3C. RAG1 and RAG2 shared enriched proteins**

| BCORL1 | COPB2 | DCAF1 |
| --- | --- | --- |
| DNAJC17 | FAM120A | FAM193B |
| FHOD1 | GALNT2 | LENG8 |
| MGST3 | MRPS31 | NCAPG2 |
| NUP37 | POLA1 | RAG1 |
| RAG2 | RB1 | RFX5 |
| SFXN4 | UBTF | WDR18 |
| ZNF281 | ZNF687 |  |

**Supplementary Table S4. RAG interactome GO analysis**

| **RAG1 CC** |  |  |  |  |  |  |
| --- | --- | --- | --- | --- | --- | --- |
| **ID** | **Description** | **GeneRatio** | **BgRatio** | **pvalue** | **p.adjust** | **qvalue** |
| GO:0035097 | histone methyltransferase complex | 4/84 | 77/19559 | 0.000337 | 0.033099 | 0.026813 |
| GO:0005667 | transcription regulator complex | 8/84 | 413/19559 | 0.000397 | 0.033099 | 0.026813 |
| GO:0090575 | RNA polymerase II transcription regulator complex | 5/84 | 161/19559 | 0.000649 | 0.033099 | 0.026813 |
| GO:0030117 | membrane coat | 4/84 | 94/19559 | 0.00072 | 0.033099 | 0.026813 |
| GO:0048475 | coated membrane | 4/84 | 94/19559 | 0.00072 | 0.033099 | 0.026813 |
| GO:0034708 | methyltransferase complex | 4/84 | 105/19559 | 0.001088 | 0.0379 | 0.030702 |
| GO:0030662 | coated vesicle membrane | 5/84 | 183/19559 | 0.001153 | 0.0379 | 0.030702 |
| GO:0030126 | COPI vesicle coat | 2/84 | 13/19559 | 0.001379 | 0.039634 | 0.032106 |
| GO:0016607 | nuclear speck | 7/84 | 401/19559 | 0.001688 | 0.039774 | 0.03222 |
| GO:0030120 | vesicle coat | 3/84 | 56/19559 | 0.001798 | 0.039774 | 0.03222 |
| GO:0032993 | protein-DNA complex | 5/84 | 205/19559 | 0.001902 | 0.039774 | 0.03222 |
| GO:0035098 | ESC/E(Z) complex | 2/84 | 16/19559 | 0.002103 | 0.040312 | 0.032655 |
| GO:0030663 | COPI-coated vesicle membrane | 2/84 | 18/19559 | 0.002667 | 0.04381 | 0.035489 |
| GO:0031143 | pseudopodium | 2/84 | 18/19559 | 0.002667 | 0.04381 | 0.035489 |
| GO:0070603 | SWI/SNF superfamily-type complex | 3/84 | 74/19559 | 0.003977 | 0.051329 | 0.04158 |
| GO:0005635 | nuclear envelope | 7/84 | 473/19559 | 0.004225 | 0.051329 | 0.04158 |
| GO:0005759 | mitochondrial matrix | 7/84 | 473/19559 | 0.004225 | 0.051329 | 0.04158 |
| GO:0030687 | preribosome, large subunit precursor | 2/84 | 23/19559 | 0.004349 | 0.051329 | 0.04158 |
| GO:0030684 | preribosome | 3/84 | 78/19559 | 0.00461 | 0.051329 | 0.04158 |
| GO:1904949 | ATPase complex | 3/84 | 78/19559 | 0.00461 | 0.051329 | 0.04158 |
| GO:0061695 | transferase complex, transferring phosphorus-containing groups | 5/84 | 253/19559 | 0.004687 | 0.051329 | 0.04158 |
| GO:0030131 | clathrin adaptor complex | 2/84 | 25/19559 | 0.005128 | 0.05361 | 0.043428 |
| GO:0030137 | COPI-coated vesicle | 2/84 | 28/19559 | 0.006408 | 0.063141 | 0.051149 |
| GO:0044665 | MLL1/2 complex | 2/84 | 29/19559 | 0.006863 | 0.063141 | 0.051149 |
| GO:0071339 | MLL1 complex | 2/84 | 29/19559 | 0.006863 | 0.063141 | 0.051149 |
| GO:0030135 | coated vesicle | 5/84 | 298/19559 | 0.009209 | 0.081462 | 0.06599 |
| GO:0055029 | nuclear DNA-directed RNA polymerase complex | 3/84 | 103/19559 | 0.009928 | 0.08284 | 0.067106 |
| GO:0000428 | DNA-directed RNA polymerase complex | 3/84 | 104/19559 | 0.010191 | 0.08284 | 0.067106 |
| GO:0008180 | COP9 signalosome | 2/84 | 36/19559 | 0.010445 | 0.08284 | 0.067106 |
| GO:1905348 | endonuclease complex | 2/84 | 37/19559 | 0.011011 | 0.083739 | 0.067834 |
| GO:0030880 | RNA polymerase complex | 3/84 | 108/19559 | 0.011287 | 0.083739 | 0.067834 |
| GO:0030660 | Golgi-associated vesicle membrane | 3/84 | 112/19559 | 0.012447 | 0.089466 | 0.072473 |
| GO:0030119 | AP-type membrane coat adaptor complex | 2/84 | 41/19559 | 0.013408 | 0.093452 | 0.075702 |
| GO:0030665 | clathrin-coated vesicle membrane | 3/84 | 118/19559 | 0.014313 | 0.096822 | 0.078432 |
| GO:0000793 | condensed chromosome | 4/84 | 222/19559 | 0.015379 | 0.101061 | 0.081866 |
| GO:0030118 | clathrin coat | 2/84 | 46/19559 | 0.016692 | 0.106642 | 0.086387 |
| GO:0031519 | PcG protein complex | 2/84 | 47/19559 | 0.017386 | 0.108072 | 0.087546 |
| GO:0005743 | mitochondrial inner membrane | 6/84 | 489/19559 | 0.018696 | 0.113159 | 0.091667 |
| GO:0014704 | intercalated disc | 2/84 | 51/19559 | 0.020281 | 0.119604 | 0.096887 |
| GO:0001725 | stress fiber | 2/84 | 68/19559 | 0.034581 | 0.19399 | 0.157145 |
| GO:0097517 | contractile actin filament bundle | 2/84 | 68/19559 | 0.034581 | 0.19399 | 0.157145 |
|  |  |  |  |  |  |  |
| **RAG2 CC** |  |  |  |  |  |  |
| **ID** | **Description** | **GeneRatio** | **BgRatio** | **pvalue** | **p.adjust** | **qvalue** |
| GO:0098687 | chromosomal region | 14/142 | 348/19550 | 2.54E-07 | 7.11E-05 | 6.01E-05 |
| GO:0005667 | transcription regulator complex | 14/142 | 413/19550 | 1.95E-06 | 0.000184 | 0.000156 |
| GO:0032993 | protein-DNA complex | 10/142 | 199/19550 | 1.97E-06 | 0.000184 | 0.000156 |
| GO:0000779 | condensed chromosome, centromeric region | 7/142 | 144/19550 | 8.82E-05 | 0.00405 | 0.003426 |
| GO:0055029 | nuclear DNA-directed RNA polymerase complex | 6/142 | 100/19550 | 8.99E-05 | 0.00405 | 0.003426 |
| GO:0000428 | DNA-directed RNA polymerase complex | 6/142 | 101/19550 | 9.5E-05 | 0.00405 | 0.003426 |
| GO:0000775 | chromosome, centromeric region | 8/142 | 198/19550 | 0.000101 | 0.00405 | 0.003426 |
| GO:0030880 | RNA polymerase complex | 6/142 | 105/19550 | 0.000118 | 0.004124 | 0.003488 |
| GO:0005635 | nuclear envelope | 12/142 | 468/19550 | 0.000161 | 0.005011 | 0.004239 |
| GO:0042575 | DNA polymerase complex | 3/142 | 19/19550 | 0.000334 | 0.009341 | 0.007901 |
| GO:0005643 | nuclear pore | 5/142 | 84/19550 | 0.000367 | 0.009341 | 0.007901 |
| GO:0000793 | condensed chromosome | 8/142 | 244/19550 | 0.000419 | 0.009772 | 0.008266 |
| GO:0001650 | fibrillar center | 6/142 | 140/19550 | 0.000558 | 0.012025 | 0.010172 |
| GO:0000314 | organellar small ribosomal subunit | 3/142 | 28/19550 | 0.001076 | 0.018828 | 0.015926 |
| GO:0005763 | mitochondrial small ribosomal subunit | 3/142 | 28/19550 | 0.001076 | 0.018828 | 0.015926 |
| GO:0043596 | nuclear replication fork | 3/142 | 28/19550 | 0.001076 | 0.018828 | 0.015926 |
| GO:0000781 | chromosome, telomeric region | 6/142 | 162/19550 | 0.001195 | 0.019679 | 0.016645 |
| GO:0000228 | nuclear chromosome | 7/142 | 227/19550 | 0.001371 | 0.021211 | 0.017942 |
| GO:0090575 | RNA polymerase II transcription regulator complex | 6/142 | 168/19550 | 0.001439 | 0.021211 | 0.017942 |
| GO:0000792 | heterochromatin | 4/142 | 73/19550 | 0.00197 | 0.027578 | 0.023327 |
| GO:0071162 | CMG complex | 2/142 | 10/19550 | 0.002269 | 0.030255 | 0.025591 |
| GO:0005759 | mitochondrial matrix | 10/142 | 480/19550 | 0.002692 | 0.031828 | 0.026922 |
| GO:0005681 | spliceosomal complex | 6/142 | 191/19550 | 0.002745 | 0.031828 | 0.026922 |
| GO:0005845 | mRNA cap binding complex | 2/142 | 11/19550 | 0.00276 | 0.031828 | 0.026922 |
| GO:0000776 | kinetochore | 5/142 | 134/19550 | 0.002973 | 0.031828 | 0.026922 |
| GO:0016607 | nuclear speck | 9/142 | 412/19550 | 0.0032 | 0.031828 | 0.026922 |
| GO:0000313 | organellar ribosome | 4/142 | 84/19550 | 0.003284 | 0.031828 | 0.026922 |
| GO:0005761 | mitochondrial ribosome | 4/142 | 84/19550 | 0.003284 | 0.031828 | 0.026922 |
| GO:0031261 | DNA replication preinitiation complex | 2/142 | 12/19550 | 0.003296 | 0.031828 | 0.026922 |
| GO:0034518 | RNA cap binding complex | 2/142 | 13/19550 | 0.003877 | 0.035021 | 0.029623 |
| GO:0043601 | nuclear replisome | 2/142 | 13/19550 | 0.003877 | 0.035021 | 0.029623 |
| GO:0030894 | replisome | 2/142 | 15/19550 | 0.00517 | 0.045238 | 0.038265 |
| GO:0061695 | transferase complex, transferring phosphorus-containing groups | 6/142 | 239/19550 | 0.008082 | 0.066092 | 0.055905 |
| GO:0005840 | ribosome | 6/142 | 240/19550 | 0.008241 | 0.066092 | 0.055905 |
| GO:0016514 | SWI/SNF complex | 2/142 | 19/19550 | 0.008261 | 0.066092 | 0.055905 |
| GO:0016363 | nuclear matrix | 4/142 | 111/19550 | 0.008774 | 0.068246 | 0.057727 |
| GO:0005657 | replication fork | 3/142 | 60/19550 | 0.009492 | 0.071834 | 0.060762 |
| GO:0044391 | ribosomal subunit | 5/142 | 180/19550 | 0.010186 | 0.07355 | 0.062214 |
| GO:0005743 | mitochondrial inner membrane | 9/142 | 495/19550 | 0.010252 | 0.07355 | 0.062214 |
| GO:0044815 | DNA packaging complex | 4/142 | 117/19550 | 0.010507 | 0.07355 | 0.062214 |
| GO:0005721 | pericentric heterochromatin | 2/142 | 22/19550 | 0.011003 | 0.075141 | 0.063559 |
| GO:0015935 | small ribosomal subunit | 3/142 | 71/19550 | 0.014967 | 0.09757 | 0.082531 |
| GO:0034399 | nuclear periphery | 4/142 | 130/19550 | 0.014984 | 0.09757 | 0.082531 |
| GO:0070603 | SWI/SNF superfamily-type complex | 3/142 | 72/19550 | 0.015536 | 0.098866 | 0.083627 |
| GO:1904949 | ATPase complex | 3/142 | 73/19550 | 0.016118 | 0.099388 | 0.084069 |
| GO:0032039 | integrator complex | 2/142 | 27/19550 | 0.016328 | 0.099388 | 0.084069 |
| GO:0030137 | COPI-coated vesicle | 2/142 | 28/19550 | 0.017501 | 0.104263 | 0.088192 |
| GO:0016591 | RNA polymerase II, holoenzyme | 3/142 | 76/19550 | 0.017935 | 0.104618 | 0.088493 |
| GO:0005669 | transcription factor TFIID complex | 2/142 | 33/19550 | 0.023877 | 0.136442 | 0.115411 |
| GO:0071013 | catalytic step 2 spliceosome | 3/142 | 87/19550 | 0.025535 | 0.142999 | 0.120958 |
| GO:0034708 | methyltransferase complex | 3/142 | 88/19550 | 0.0263 | 0.144391 | 0.122135 |
| GO:0005684 | U2-type spliceosomal complex | 3/142 | 92/19550 | 0.029479 | 0.15873 | 0.134264 |
| GO:1990391 | DNA repair complex | 2/142 | 39/19550 | 0.032579 | 0.172116 | 0.145587 |
|  |  |  |  |  |  |  |
| **RAG1 BP** |  |  |  |  |  |  |
| **ID** | **Description** | **GeneRatio** | **BgRatio** | **pvalue** | **p.adjust** | **qvalue** |
| GO:0006364 | rRNA processing | 8/81 | 227/18866 | 5.87E-06 | 0.006152 | 0.005803 |
| GO:0016072 | rRNA metabolic process | 8/81 | 238/18866 | 8.3E-06 | 0.006152 | 0.005803 |
| GO:0033151 | V(D)J recombination | 3/81 | 15/18866 | 3.34E-05 | 0.016355 | 0.015426 |
| GO:0034470 | ncRNA processing | 9/81 | 400/18866 | 5.37E-05 | 0.016355 | 0.015426 |
| GO:0042254 | ribosome biogenesis | 8/81 | 310/18866 | 5.52E-05 | 0.016355 | 0.015426 |
| GO:0000470 | maturation of LSU-rRNA | 3/81 | 28/18866 | 0.000231 | 0.05501 | 0.051888 |
| GO:0034660 | ncRNA metabolic process | 9/81 | 493/18866 | 0.00026 | 0.05501 | 0.051888 |
| GO:0000083 | regulation of transcription involved in G1/S transition of mitotic cell cycle | 3/81 | 35/18866 | 0.000452 | 0.083723 | 0.078972 |
| GO:0070317 | negative regulation of G0 to G1 transition | 3/81 | 41/18866 | 0.000723 | 0.106397 | 0.100359 |
| GO:0002327 | immature B cell differentiation | 2/81 | 10/18866 | 0.000801 | 0.106397 | 0.100359 |
| GO:0016570 | histone modification | 8/81 | 468/18866 | 0.000891 | 0.106397 | 0.100359 |
| GO:0070316 | regulation of G0 to G1 transition | 3/81 | 45/18866 | 0.00095 | 0.106397 | 0.100359 |
| GO:0045023 | G0 to G1 transition | 3/81 | 46/18866 | 0.001013 | 0.106397 | 0.100359 |
| GO:0016569 | covalent chromatin modification | 8/81 | 481/18866 | 0.001063 | 0.106397 | 0.100359 |
| GO:0022613 | ribonucleoprotein complex biogenesis | 8/81 | 482/18866 | 0.001077 | 0.106397 | 0.100359 |
| GO:0051639 | actin filament network formation | 2/81 | 12/18866 | 0.001169 | 0.108239 | 0.102097 |
| GO:0034968 | histone lysine methylation | 4/81 | 116/18866 | 0.001568 | 0.136706 | 0.128948 |
| GO:1904263 | positive regulation of TORC1 signaling | 2/81 | 17/18866 | 0.002375 | 0.186203 | 0.175636 |
| GO:0006338 | chromatin remodeling | 5/81 | 218/18866 | 0.002474 | 0.186203 | 0.175636 |
| GO:0018022 | peptidyl-lysine methylation | 4/81 | 132/18866 | 0.002513 | 0.186203 | 0.175636 |
| GO:0002562 | somatic diversification of immune receptors via germline recombination within a single locus | 3/81 | 66/18866 | 0.002872 | 0.193465 | 0.182486 |
| GO:0016444 | somatic cell DNA recombination | 3/81 | 66/18866 | 0.002872 | 0.193465 | 0.182486 |
|  |  |  |  |  |  |  |
| **RAG2 BP** |  |  |  |  |  |  |
| **ID** | **Description** | **GeneRatio** | **BgRatio** | **pvalue** | **p.adjust** | **qvalue** |
| GO:0006310 | DNA recombination | 13/136 | 305/18723 | 3.55E-07 | 0.00068 | 0.000602 |
| GO:0051168 | nuclear export | 9/136 | 154/18723 | 1.88E-06 | 0.001794 | 0.001588 |
| GO:0016570 | histone modification | 14/136 | 463/18723 | 7.14E-06 | 0.003909 | 0.003459 |
| GO:0051052 | regulation of DNA metabolic process | 12/136 | 359/18723 | 1.24E-05 | 0.003909 | 0.003459 |
| GO:0006913 | nucleocytoplasmic transport | 11/136 | 301/18723 | 1.27E-05 | 0.003909 | 0.003459 |
| GO:0051169 | nuclear transport | 11/136 | 301/18723 | 1.27E-05 | 0.003909 | 0.003459 |
| GO:0006403 | RNA localization | 9/136 | 201/18723 | 1.64E-05 | 0.003909 | 0.003459 |
| GO:0071426 | ribonucleoprotein complex export from nucleus | 6/136 | 76/18723 | 1.89E-05 | 0.003909 | 0.003459 |
| GO:0002200 | somatic diversification of immune receptors | 6/136 | 77/18723 | 2.04E-05 | 0.003909 | 0.003459 |
| GO:0071166 | ribonucleoprotein complex localization | 6/136 | 77/18723 | 2.04E-05 | 0.003909 | 0.003459 |
| GO:0050657 | nucleic acid transport | 8/136 | 163/18723 | 2.54E-05 | 0.004059 | 0.003591 |
| GO:0050658 | RNA transport | 8/136 | 163/18723 | 2.54E-05 | 0.004059 | 0.003591 |
| GO:0051236 | establishment of RNA localization | 8/136 | 166/18723 | 2.9E-05 | 0.00427 | 0.003779 |
| GO:0006405 | RNA export from nucleus | 6/136 | 84/18723 | 3.36E-05 | 0.004592 | 0.004063 |
| GO:0071103 | DNA conformation change | 10/136 | 290/18723 | 5.2E-05 | 0.006433 | 0.005692 |
| GO:0034660 | ncRNA metabolic process | 13/136 | 485/18723 | 5.38E-05 | 0.006433 | 0.005692 |
| GO:0006406 | mRNA export from nucleus | 5/136 | 59/18723 | 6.86E-05 | 0.00725 | 0.006415 |
| GO:0071427 | mRNA-containing ribonucleoprotein complex export from nucleus | 5/136 | 59/18723 | 6.86E-05 | 0.00725 | 0.006415 |
| GO:0016577 | histone demethylation | 4/136 | 31/18723 | 7.2E-05 | 0.00725 | 0.006415 |
| GO:0006482 | protein demethylation | 4/136 | 33/18723 | 9.25E-05 | 0.008435 | 0.007464 |
| GO:0008214 | protein dealkylation | 4/136 | 33/18723 | 9.25E-05 | 0.008435 | 0.007464 |
| GO:0048820 | hair follicle maturation | 3/136 | 13/18723 | 0.000102 | 0.008679 | 0.00768 |
| GO:0006270 | DNA replication initiation | 4/136 | 34/18723 | 0.000104 | 0.008679 | 0.00768 |
| GO:0002562 | somatic diversification of immune receptors via germline recombination within a single locus | 5/136 | 66/18723 | 0.000118 | 0.009007 | 0.00797 |
| GO:0016444 | somatic cell DNA recombination | 5/136 | 66/18723 | 0.000118 | 0.009007 | 0.00797 |
| GO:1902969 | mitotic DNA replication | 3/136 | 14/18723 | 0.000129 | 0.009237 | 0.008174 |
| GO:0000018 | regulation of DNA recombination | 6/136 | 107/18723 | 0.00013 | 0.009237 | 0.008174 |
| GO:0034470 | ncRNA processing | 11/136 | 395/18723 | 0.000148 | 0.010105 | 0.008942 |
| GO:0070988 | demethylation | 5/136 | 73/18723 | 0.00019 | 0.012531 | 0.011088 |
| GO:0015931 | nucleobase-containing compound transport | 8/136 | 222/18723 | 0.000221 | 0.013745 | 0.012163 |
| GO:0051101 | regulation of DNA binding | 6/136 | 118/18723 | 0.000223 | 0.013745 | 0.012163 |
| GO:0033151 | V(D)J recombination | 3/136 | 17/18723 | 0.000237 | 0.013751 | 0.012168 |
| GO:0000075 | cell cycle checkpoint signaling | 7/136 | 169/18723 | 0.000237 | 0.013751 | 0.012168 |
| GO:0051098 | regulation of binding | 10/136 | 363/18723 | 0.000324 | 0.018159 | 0.016068 |
| GO:0008380 | RNA splicing | 11/136 | 434/18723 | 0.000332 | 0.018159 | 0.016068 |
| GO:0032508 | DNA duplex unwinding | 5/136 | 84/18723 | 0.000366 | 0.018904 | 0.016728 |
| GO:0045911 | positive regulation of DNA recombination | 4/136 | 47/18723 | 0.000373 | 0.018904 | 0.016728 |
| GO:0051028 | mRNA transport | 6/136 | 130/18723 | 0.000375 | 0.018904 | 0.016728 |
| GO:0030261 | chromosome condensation | 4/136 | 48/18723 | 0.000404 | 0.019852 | 0.017567 |
| GO:0033044 | regulation of chromosome organization | 7/136 | 187/18723 | 0.000438 | 0.02094 | 0.01853 |
| GO:1901988 | negative regulation of cell cycle phase transition | 8/136 | 249/18723 | 0.000477 | 0.021939 | 0.019413 |
| GO:0006302 | double-strand break repair | 8/136 | 251/18723 | 0.000502 | 0.021939 | 0.019413 |
| GO:0032392 | DNA geometric change | 5/136 | 90/18723 | 0.000503 | 0.021939 | 0.019413 |
| GO:0043414 | macromolecule methylation | 9/136 | 316/18723 | 0.00051 | 0.021939 | 0.019413 |
| GO:0000724 | double-strand break repair via homologous recombination | 6/136 | 138/18723 | 0.000516 | 0.021939 | 0.019413 |
| GO:0000725 | recombinational repair | 6/136 | 140/18723 | 0.000557 | 0.023139 | 0.020475 |
| GO:1901987 | regulation of cell cycle phase transition | 10/136 | 390/18723 | 0.000568 | 0.023139 | 0.020475 |
| GO:1901796 | regulation of signal transduction by p53 class mediator | 5/136 | 93/18723 | 0.000585 | 0.023331 | 0.020645 |
| GO:0051054 | positive regulation of DNA metabolic process | 7/136 | 201/18723 | 0.000673 | 0.025989 | 0.022998 |
| GO:0006360 | transcription by RNA polymerase I | 4/136 | 55/18723 | 0.000682 | 0.025989 | 0.022998 |
| GO:0000819 | sister chromatid segregation | 7/136 | 202/18723 | 0.000693 | 0.025989 | 0.022998 |
| GO:0043388 | positive regulation of DNA binding | 4/136 | 56/18723 | 0.00073 | 0.026872 | 0.023779 |
| GO:0006325 | chromatin organization | 10/136 | 409/18723 | 0.000819 | 0.029343 | 0.025965 |
| GO:0006261 | DNA-dependent DNA replication | 6/136 | 151/18723 | 0.000828 | 0.029343 | 0.025965 |
| GO:0007059 | chromosome segregation | 9/136 | 346/18723 | 0.00097 | 0.033768 | 0.029881 |
| GO:0070076 | histone lysine demethylation | 3/136 | 28/18723 | 0.001075 | 0.036428 | 0.032235 |
| GO:0042303 | molting cycle | 5/136 | 107/18723 | 0.001104 | 0.036428 | 0.032235 |
| GO:0042633 | hair cycle | 5/136 | 107/18723 | 0.001104 | 0.036428 | 0.032235 |
| GO:0031503 | protein-containing complex localization | 7/136 | 220/18723 | 0.00114 | 0.036991 | 0.032733 |
| GO:0072331 | signal transduction by p53 class mediator | 6/136 | 163/18723 | 0.00123 | 0.039225 | 0.03471 |
| GO:0032259 | methylation | 9/136 | 364/18723 | 0.00138 | 0.042428 | 0.037545 |
| GO:0009451 | RNA modification | 6/136 | 167/18723 | 0.001392 | 0.042428 | 0.037545 |
| GO:0010948 | negative regulation of cell cycle process | 8/136 | 294/18723 | 0.001397 | 0.042428 | 0.037545 |
| GO:0000070 | mitotic sister chromatid segregation | 6/136 | 168/18723 | 0.001435 | 0.042755 | 0.037834 |
| GO:0071168 | protein localization to chromatin | 3/136 | 31/18723 | 0.001452 | 0.042755 | 0.037834 |
| GO:0042254 | ribosome biogenesis | 8/136 | 299/18723 | 0.001553 | 0.045047 | 0.039862 |
| GO:0051099 | positive regulation of binding | 6/136 | 173/18723 | 0.001666 | 0.0476 | 0.042121 |
|  |  |  |  |  |  |  |
| **RAG1 MF** |  |  |  |  |  |  |
| **ID** | **Description** | **GeneRatio** | **BgRatio** | **pvalue** | **p.adjust** | **qvalue** |
| GO:0042393 | histone binding | 9/80 | 238/18352 | 9.29E-07 | 0.000216 | 0.00018 |
| GO:0140030 | modification-dependent protein binding | 6/80 | 153/18352 | 5.5E-05 | 0.006382 | 0.005328 |
| GO:0051536 | iron-sulfur cluster binding | 4/80 | 67/18352 | 0.000208 | 0.009024 | 0.007534 |
| GO:0051540 | metal cluster binding | 4/80 | 67/18352 | 0.000208 | 0.009024 | 0.007534 |
| GO:0035064 | methylated histone binding | 4/80 | 69/18352 | 0.000233 | 0.009024 | 0.007534 |
| GO:0140034 | methylation-dependent protein binding | 4/80 | 69/18352 | 0.000233 | 0.009024 | 0.007534 |
| GO:0051219 | phosphoprotein binding | 4/80 | 85/18352 | 0.000519 | 0.017186 | 0.014347 |
| GO:0140223 | general transcription initiation factor activity | 3/80 | 43/18352 | 0.000868 | 0.023942 | 0.019988 |
| GO:0018024 | histone-lysine N-methyltransferase activity | 3/80 | 44/18352 | 0.000929 | 0.023942 | 0.019988 |
| GO:0001046 | core promoter sequence-specific DNA binding | 3/80 | 46/18352 | 0.001058 | 0.024543 | 0.02049 |
| GO:0042054 | histone methyltransferase activity | 3/80 | 59/18352 | 0.002175 | 0.044743 | 0.037354 |
| GO:0016279 | protein-lysine N-methyltransferase activity | 3/80 | 61/18352 | 0.002393 | 0.044743 | 0.037354 |
| GO:0016278 | lysine N-methyltransferase activity | 3/80 | 62/18352 | 0.002507 | 0.044743 | 0.037354 |
| GO:0140297 | DNA-binding transcription factor binding | 6/80 | 347/18352 | 0.004065 | 0.059399 | 0.049589 |
| GO:0070577 | lysine-acetylated histone binding | 2/80 | 22/18352 | 0.004096 | 0.059399 | 0.049589 |
| GO:0140033 | acetylation-dependent protein binding | 2/80 | 22/18352 | 0.004096 | 0.059399 | 0.049589 |
| GO:0043425 | bHLH transcription factor binding | 2/80 | 29/18352 | 0.007059 | 0.094842 | 0.079179 |
| GO:0008276 | protein methyltransferase activity | 3/80 | 91/18352 | 0.007358 | 0.094842 | 0.079179 |
| GO:0051721 | protein phosphatase 2A binding | 2/80 | 32/18352 | 0.008552 | 0.104421 | 0.087175 |
| GO:0008170 | N-methyltransferase activity | 3/80 | 100/18352 | 0.009525 | 0.110487 | 0.092239 |
| GO:0008144 | drug binding | 3/80 | 104/18352 | 0.010595 | 0.111072 | 0.092728 |
| GO:0016251 | RNA polymerase II general transcription initiation factor activity | 2/80 | 37/18352 | 0.011323 | 0.111072 | 0.092728 |
| GO:0003755 | peptidyl-prolyl cis-trans isomerase activity | 2/80 | 38/18352 | 0.011918 | 0.111072 | 0.092728 |
| GO:0044389 | ubiquitin-like protein ligase binding | 5/80 | 316/18352 | 0.012326 | 0.111072 | 0.092728 |
| GO:0016409 | palmitoyltransferase activity | 2/80 | 39/18352 | 0.012527 | 0.111072 | 0.092728 |
| GO:0004197 | cysteine-type endopeptidase activity | 3/80 | 114/18352 | 0.013564 | 0.111072 | 0.092728 |
| GO:0016859 | cis-trans isomerase activity | 2/80 | 41/18352 | 0.013785 | 0.111072 | 0.092728 |
| GO:0004843 | thiol-dependent ubiquitin-specific protease activity | 3/80 | 115/18352 | 0.013884 | 0.111072 | 0.092728 |
| GO:0101005 | ubiquitinyl hydrolase activity | 3/80 | 115/18352 | 0.013884 | 0.111072 | 0.092728 |
| GO:0051539 | 4 iron, 4 sulfur cluster binding | 2/80 | 42/18352 | 0.014434 | 0.111623 | 0.093188 |
| GO:0008242 | omega peptidase activity | 3/80 | 128/18352 | 0.018441 | 0.138014 | 0.11522 |
| GO:0008234 | cysteine-type peptidase activity | 3/80 | 139/18352 | 0.022873 | 0.165827 | 0.13844 |
| GO:0035257 | nuclear hormone receptor binding | 3/80 | 144/18352 | 0.025062 | 0.176194 | 0.147095 |
| GO:1990841 | promoter-specific chromatin binding | 2/80 | 58/18352 | 0.026499 | 0.18082 | 0.150956 |
| GO:0097110 | scaffold protein binding | 2/80 | 60/18352 | 0.028217 | 0.187041 | 0.15615 |
| GO:0061629 | RNA polymerase II-specific DNA-binding transcription factor binding | 4/80 | 267/18352 | 0.029297 | 0.188806 | 0.157624 |
| GO:0016853 | isomerase activity | 3/80 | 156/18352 | 0.030762 | 0.192886 | 0.16103 |
|  |  |  |  |  |  |  |
| **RAG2 MF** |  |  |  |  |  |  |
| **ID** | **Description** | **GeneRatio** | **BgRatio** | **pvalue** | **p.adjust** | **qvalue** |
| GO:0042393 | histone binding | 12/142 | 244/18368 | 4.37E-07 | 0.000145 | 0.000122 |
| GO:0008022 | protein C-terminus binding | 10/142 | 189/18368 | 2.15E-06 | 0.000357 | 0.0003 |
| GO:1990841 | promoter-specific chromatin binding | 6/142 | 62/18368 | 8.27E-06 | 0.000915 | 0.000769 |
| GO:0140098 | catalytic activity, acting on RNA | 12/142 | 386/18368 | 4.68E-05 | 0.003872 | 0.003253 |
| GO:0032452 | histone demethylase activity | 4/142 | 29/18368 | 7E-05 | 0.003872 | 0.003253 |
| GO:0140457 | protein demethylase activity | 4/142 | 29/18368 | 7E-05 | 0.003872 | 0.003253 |
| GO:0008094 | ATP-dependent activity, acting on DNA | 6/142 | 111/18368 | 0.000223 | 0.007648 | 0.006426 |
| GO:0032451 | demethylase activity | 4/142 | 39/18368 | 0.000228 | 0.007648 | 0.006426 |
| GO:0000049 | tRNA binding | 5/142 | 72/18368 | 0.000238 | 0.007648 | 0.006426 |
| GO:0035064 | methylated histone binding | 5/142 | 73/18368 | 0.000253 | 0.007648 | 0.006426 |
| GO:0140034 | methylation-dependent protein binding | 5/142 | 73/18368 | 0.000253 | 0.007648 | 0.006426 |
| GO:0140097 | catalytic activity, acting on DNA | 8/142 | 229/18368 | 0.000414 | 0.011451 | 0.009621 |
| GO:0061629 | RNA polymerase II-specific DNA-binding transcription factor binding | 9/142 | 299/18368 | 0.000537 | 0.01372 | 0.011527 |
| GO:0140297 | DNA-binding transcription factor binding | 10/142 | 394/18368 | 0.000993 | 0.023546 | 0.019783 |
| GO:0140030 | modification-dependent protein binding | 6/142 | 160/18368 | 0.001537 | 0.034013 | 0.028578 |
| GO:0019843 | rRNA binding | 4/142 | 68/18368 | 0.001901 | 0.039439 | 0.033137 |
| GO:0003678 | DNA helicase activity | 4/142 | 72/18368 | 0.002345 | 0.043879 | 0.036867 |
| GO:0008097 | 5S rRNA binding | 2/142 | 10/18368 | 0.002564 | 0.043879 | 0.036867 |
| GO:0097718 | disordered domain specific binding | 3/142 | 36/18368 | 0.00268 | 0.043879 | 0.036867 |
| GO:0003697 | single-stranded DNA binding | 5/142 | 123/18368 | 0.00268 | 0.043879 | 0.036867 |
| GO:0140101 | catalytic activity, acting on a tRNA | 5/142 | 124/18368 | 0.002775 | 0.043879 | 0.036867 |
| GO:0042826 | histone deacetylase binding | 5/142 | 127/18368 | 0.003077 | 0.046433 | 0.039013 |
| GO:0004402 | histone acetyltransferase activity | 3/142 | 39/18368 | 0.003373 | 0.048681 | 0.040902 |
| GO:0008174 | mRNA methyltransferase activity | 2/142 | 12/18368 | 0.003723 | 0.051501 | 0.043271 |
| GO:0061733 | peptide-lysine-N-acetyltransferase activity | 3/142 | 41/18368 | 0.00389 | 0.051657 | 0.043402 |
| GO:0051539 | 4 iron, 4 sulfur cluster binding | 3/142 | 42/18368 | 0.004166 | 0.051906 | 0.043611 |
| GO:0003713 | transcription coactivator activity | 7/142 | 262/18368 | 0.004313 | 0.051906 | 0.043611 |
| GO:0032454 | histone H3-methyl-lysine-9 demethylase activity | 2/142 | 13/18368 | 0.004378 | 0.051906 | 0.043611 |
| GO:0003727 | single-stranded RNA binding | 4/142 | 87/18368 | 0.004641 | 0.053134 | 0.044643 |
| GO:0016922 | nuclear receptor binding | 5/142 | 144/18368 | 0.005247 | 0.056522 | 0.04749 |
| GO:0016887 | ATP hydrolysis activity | 7/142 | 272/18368 | 0.005278 | 0.056522 | 0.04749 |
| GO:0034212 | peptide N-acetyltransferase activity | 3/142 | 49/18368 | 0.006428 | 0.064702 | 0.054363 |
| GO:0008168 | methyltransferase activity | 6/142 | 214/18368 | 0.006431 | 0.064702 | 0.054363 |
| GO:0035173 | histone kinase activity | 2/142 | 16/18368 | 0.006633 | 0.064773 | 0.054422 |
| GO:0004386 | helicase activity | 5/142 | 156/18368 | 0.007316 | 0.067472 | 0.05669 |
| GO:0008757 | S-adenosylmethionine-dependent methyltransferase activity | 5/142 | 156/18368 | 0.007316 | 0.067472 | 0.05669 |
| GO:0016741 | transferase activity, transferring one-carbon groups | 6/142 | 225/18368 | 0.008141 | 0.073052 | 0.061378 |
| GO:0070182 | DNA polymerase binding | 2/142 | 22/18368 | 0.012389 | 0.108237 | 0.090941 |
| GO:0017116 | single-stranded DNA helicase activity | 2/142 | 23/18368 | 0.0135 | 0.110164 | 0.092561 |
| GO:0003735 | structural constituent of ribosome | 5/142 | 182/18368 | 0.013628 | 0.110164 | 0.092561 |
| GO:0003712 | transcription coregulator activity | 9/142 | 489/18368 | 0.013839 | 0.110164 | 0.092561 |
| GO:0008173 | RNA methyltransferase activity | 3/142 | 65/18368 | 0.013936 | 0.110164 | 0.092561 |
| GO:0008080 | N-acetyltransferase activity | 3/142 | 68/18368 | 0.015725 | 0.116017 | 0.097478 |
| GO:0051536 | iron-sulfur cluster binding | 3/142 | 68/18368 | 0.015725 | 0.116017 | 0.097478 |
| GO:0051540 | metal cluster binding | 3/142 | 68/18368 | 0.015725 | 0.116017 | 0.097478 |
| GO:0016866 | intramolecular transferase activity | 2/142 | 27/18368 | 0.018357 | 0.12967 | 0.108949 |
| GO:0043425 | bHLH transcription factor binding | 2/142 | 27/18368 | 0.018357 | 0.12967 | 0.108949 |
| GO:0017056 | structural constituent of nuclear pore | 2/142 | 28/18368 | 0.01967 | 0.136051 | 0.114311 |
| GO:0008175 | tRNA methyltransferase activity | 2/142 | 32/18368 | 0.025298 | 0.17141 | 0.144019 |
| GO:0050660 | flavin adenine dinucleotide binding | 3/142 | 85/18368 | 0.028204 | 0.187276 | 0.15735 |
| GO:0140677 | molecular function activator activity | 4/142 | 150/18368 | 0.029183 | 0.189973 | 0.159616 |
